# Lactate‐Induced K370 Lactylation of STING Inhibits STING‐TBK1 Signaling and Dampens Anti‐Tumor Immunity

**DOI:** 10.1002/advs.77209

**Published:** 2026-08-15

**Authors:** Yueyao Wu, Jingzhe Wang, Xu Chen, Yuntong Yang, Ping Wei, Han Xie, Honghao Wang, Chunyu Zhang, Siyi Xu, Qi Wang, Chunlong Zhong, Jing Zhang

**Affiliations:** ^1^ Department of Neurosurgery Shanghai East Hospital School of Medicine Tongji University Shanghai China; ^2^ State Key Laboratory of Cardiovascular Diseases and Medical Innovation Center Shanghai East Hospital School of Medicine Tongji University Shanghai China

**Keywords:** STING signaling, PD‐1 blockade, phosphorylation, lactylation, LDHA inhibition

## Abstract

The stimulator of interferon genes (STING) pathway represents a central component of innate anti‐tumor immunity; however, the efficacy of STING‐targeted therapies is frequently limited by tumor‐intrinsic resistance mechanisms. Here, we identify lactate‐driven lactylation of STING at lysine 370 (K370), a primate‐conserved site, as a metabolic checkpoint restraining STING activation. Mechanistically, K370 lactylation directly weakens STING‐TBK1 interaction. In parallel, K370 lactylation rewires STING ubiquitin linkage preference by increasing K48‐ and decreasing K63‐linked ubiquitination, thereby limiting protein stability and oligomerization. Moreover, K370 lactylation weakens the interaction between STING and the COPII component SEC24A, impairing ER‐to‐Golgi trafficking—a spatial step that normally amplifies STING‐TBK1 phosphorylation and downstream signaling cascades. Conversely, inhibition of lactate production diminishes K370 lactylation, restores STING‐TBK1 association, stabilizes STING, and permits efficient ER‐to‐Golgi translocation, thereby enabling robust type I interferon signaling upon pathway stimulation. Pharmacological LDHA inhibition potentiates STING signaling and enhances the therapeutic efficacy of STING agonism alone or in combination with PD‐1 blockade in patient‐derived tumor models and orthotopic glioblastoma mouse models. In conclusion, our findings identify STING K370 lactylation as a metabolic regulatory node restraining STING signaling and provide a rationale for combining lactate‐targeted metabolic intervention with STING‐based immunotherapy.

## Graphical abstract

Lactate accumulation in glioblastoma drives lactylation of STING at lysine 370, a primate‐conserved residue. K370 lactylation weakens STING‐TBK1 binding, destabilizes STING, and disrupts ER‐to‐Golgi trafficking, blunting type I interferon signaling. LDHA inhibition reverses K370 lactylation and improves the antitumor efficacy of STING agonism combined with PD‐1 blockade in patient‐derived and orthotopic glioblastoma models.

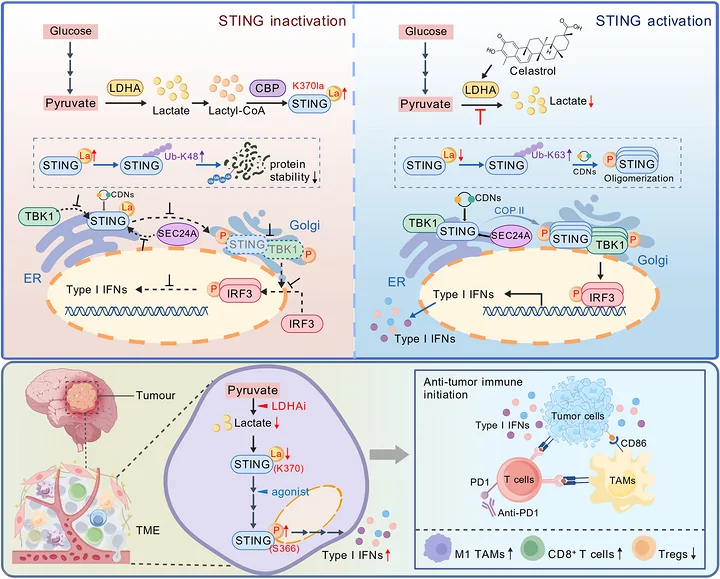

## 1 Introduction

The STING pathway serves as a central signal transducer in anti‐tumor immunity [1, 2, 3]. It can be triggered by cyclic dinucleotides (CDNs) or synthetic STING agonists that mimic CDNs [4, 5]. Emerging evidence suggests that STING pathway activation shows potent anti‐tumor activity in preclinical models, including models of glioblastoma (GBM) [6, 7]. Notably, SR‐717, a next‐generation STING agonist developed by Scripps, has been reported to robustly activate STING signaling in GBM cell lines [8, 9, 10]. In murine models of GBM, STING activation exerts anti‐tumor effects through CD8^+^ T cell‐dependent immune responses [6, 7, 11]. However, although robust anti‐tumor responses have been observed in animal models, STING agonists have shown limited efficacy in early clinical trials [5, 12]. These findings suggest that the mechanisms limiting STING activation in human cancers are not yet fully understood.

The clinical application of STING agonists in solid tumors remains challenging. First, the amino acid sequence and structural characteristics of human STING differ from those of mice and other commonly used preclinical models, resulting in species‐specific differences in ligand recognition, protein interactions, and downstream signaling [13, 14, 15]. Second, the tumor‐intrinsic and microenvironmental mechanisms that limit STING activation in solid tumors are still not fully understood [16, 17, 18]. In GBM, increased aerobic glycolysis promotes the development of an immunosuppressive tumor microenvironment [19, 20]. This metabolic reprogramming is largely driven by LDHA, which converts pyruvate to lactate, generating L‐lactate as the major lactate isoform [21, 22]. Lactate is not only a metabolic byproduct but also an immunosuppressive metabolite in the tumor microenvironment that impairs T‐cell function and promotes the accumulation of regulatory immune cells [22, 23]. In addition, lactate can induce lysine lactylation, a recently identified post‐translational modification that regulates protein stability and function [24, 25]. These findings indicate that therapeutic modulation of lactate metabolism may provide a promising approach to alleviate immunotherapy resistance in GBM [22, 26]. Intriguingly, STING has also been reported to suppress aerobic glycolysis and lactate production [27]. However, whether lactate regulates STING signaling in cancer is still unclear. In addition, the molecular mechanisms by which lactate affects human STING activation have not been defined.

In this study, we found that STING undergoes lactate‐induced lactylation at K370, a site mainly conserved in primates, and that this modification inhibits STING signaling. K370 lactylation reduces the interaction between STING and TBK1 and shifts ubiquitin linkage from K63‐ to K48‐linked ubiquitination, leading to decreased protein stability and impaired oligomerization. It also inhibits COPII‐dependent ER‐to‐Golgi trafficking of STING, a process required for efficient STING phosphorylation and downstream signal activation. In addition, targeted reconstitution experiments show that K370 lactylation disrupts STING‐TBK1 interaction independently of STING subcellular localization, indicating a direct effect of this modification on protein complex formation. By integrating human GBM cell models, patient‐derived organoids that preserve tumor microenvironmental features, and an orthotopic GL261‐hSTING glioblastoma model, we demonstrate the functional and therapeutic relevance of this regulatory axis. Importantly, pharmacologic suppression of lactate production using the LDHA inhibitor Celastrol [28, 29, 30] reduces STING lactylation, restores STING pathway responsiveness, and enhances the efficacy of STING agonists. In addition, targeting lactate metabolism enhanced STING‐mediated antitumor immunity and increased the response to STING agonist plus PD‐1 blockade therapy in both patient‐derived organoids and animal models. Together, our findings define a primate‐relevant metabolic checkpoint linking tumor lactate accumulation and protein lactylation to the suppression of innate immune signaling, providing mechanistic insight and a translational framework for improving STING‐based cancer immunotherapy.

## 2 Results

### 2.1 Lactate Modulates STING Signaling in Glioblastoma

**FIGURE 1:**
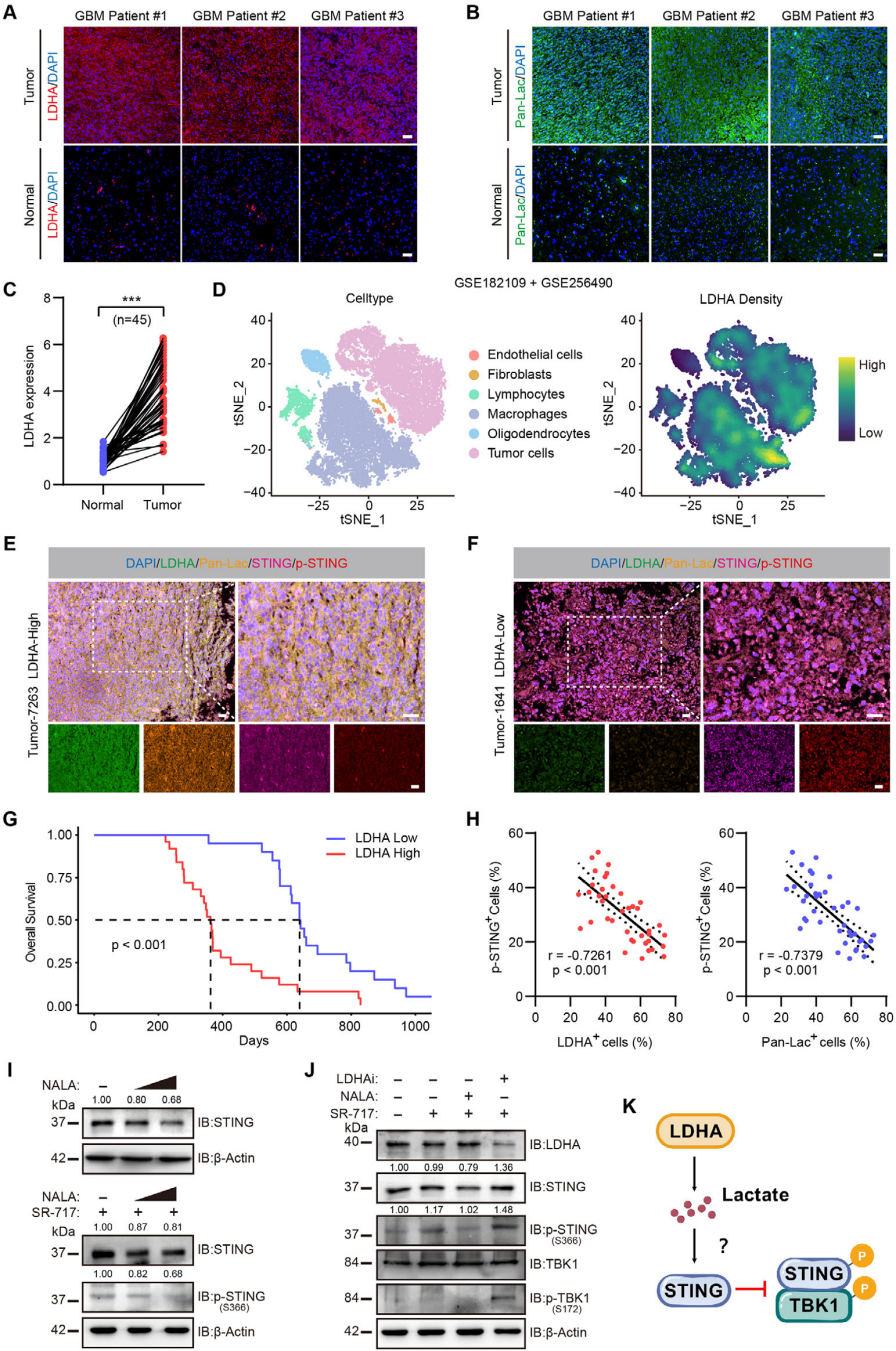
Elevated lactate is associated with impaired STING activation in glioblastoma. (A, B) Representative immunofluorescence images of LDHA and Pan‐Lac in GBM tumor tissues and normal brain tissues. Scale bar: 50 µm. (C) Quantification of LDHA immunofluorescence intensity in GBM tumor tissues and normal brain tissues (*n* = 45). *P*‐values by two‐tailed t‐test. ^***^ *p* < 0.001. (D) UMAP visualization showing LDHA expression across distinct cell‐type clusters in GSE182109 and GSE256490 single‐cell RNA‐seq datasets. (E, F) Representative immunofluorescence images of LDHA, Pan‐Lac, STING, and p‐STING (S366) in GBM tumor tissues. Scale bar: 25 µm. (G) Kaplan‐Meier analysis of the overall survival (OS) comparing high and low LDHA expression in GBM patients (*n* = 45). Patients were stratified into two groups according to the median LDHA expression level. *P*‐values by the log‐rank test. (H) Pearson correlation analysis between p‐STING and LDHA or Pan‐Lac expression in GBM tumor tissues (*n*  =  36). (I) Western blot analysis of STING expression in U251 cells treated with increasing concentrations of NALA (15 and 25 mM) in the absence of SR‐717, and of STING and p‐STING (S366) expression in cells stimulated with SR‐717 (10 µM) for 24 h. (J) Western blot analysis of STING downstream signaling in U251 cells treated with NALA (25 mM) or the LDHA inhibitor (LDHAi, OXA, 15 mM) in the presence of SR‐717 (10 µM) for 24 h. (K) Proposed model illustrating lactate‐mediated suppression of STING activation. This figure was created using BioGDP.com.

Aerobic glycolysis is a common metabolic feature of tumor cells [21, 31]. During this metabolic process, LDHA catalyzes the conversion of pyruvate to L‐lactate, contributing directly to lactate accumulation in the tumor microenvironment [21, 22]. Immunofluorescence analysis of paired glioblastoma (GBM) tumor specimens and adjacent normal tissues revealed significantly higher LDHA expression in tumors (Figure 1A–C). To further define the cellular distribution of LDHA within the GBM microenvironment, we analyzed publicly available single‐cell RNA‐sequencing datasets. Single‐cell analysis showed that LDHA expression was detected across multiple cell populations, with relatively higher expression in tumor cells and tumor‐associated macrophages (TAMs), whereas lymphocytes and oligodendrocytes displayed lower LDHA expression (Figure 1D). These findings suggest that both tumor cells and TAMs may contribute to lactate accumulation in the GBM microenvironment. Given the important role of lactate in tumor metabolism, we next assessed overall protein lactylation in GBM tissues. Immunofluorescence analysis with a pan‐lactylation (Pan‐Lac) antibody showed a marked increase in global lactylation abundance in GBM tumors compared with their paired non‐tumor tissues (Figure 1B). Consistently, Survival analysis showed that overall survival was significantly reduced in patients with high LDHA expression compared with those expressing lower levels of LDHA (Figure 1G; Figure S1A,B). In addition, GBM samples with elevated LDHA expression exhibited increased overall lactylation together with reduced STING S366 phosphorylation relative to LDHA‐low tumors (Figure 1E–H; Figure S1E). Correlation analyses further revealed significant inverse correlations between LDHA expression and p‐STING levels, as well as between Pan‐Lac signals and p‐STING levels (Figure 1H). Moreover, immunofluorescence staining of STING and p‐STING in paired tumor and normal tissues showed an overall reduction of both total STING and activated p‐STING in GBM, with a decreased p‐STING/STING ratio, consistent with impaired STING activation in tumor cells (Figure S1C,D). High‐LDHA GBM samples were associated with elevated expression of proliferation markers and immunosuppressive signatures, consistent with a more aggressive tumor phenotype (Figure S1G–J).

Building upon these clinical observations, we postulated that lactate modulates STING signaling in GBM cells. To test this, cells were treated with sodium lactate (NALA) and sodium oxamate (OXA). NALA mimics lactate functions without affecting intracellular lactate metabolism, while OXA is an LDHA inhibitor (LDHAi). Increasing concentrations of NALA caused a dose‐dependent decrease in both total STING protein and its phosphorylated form (Figure 1I). In contrast, pharmacologic inhibition of lactate production led to a robust increase in STING abundance, with a concurrent increase in phosphorylation of STING and its downstream kinase TANK‐binding kinase 1 (TBK1) (Figure 1J; Figure S1F). These findings suggest that lactate modulates STING activation and functional status through lactate‐dependent mechanisms (Figure 1K).

### 2.2 Identification of K370 as the Principal Lactylation Site on STING and CBP as Its Lactyltransferase

**FIGURE 2:**
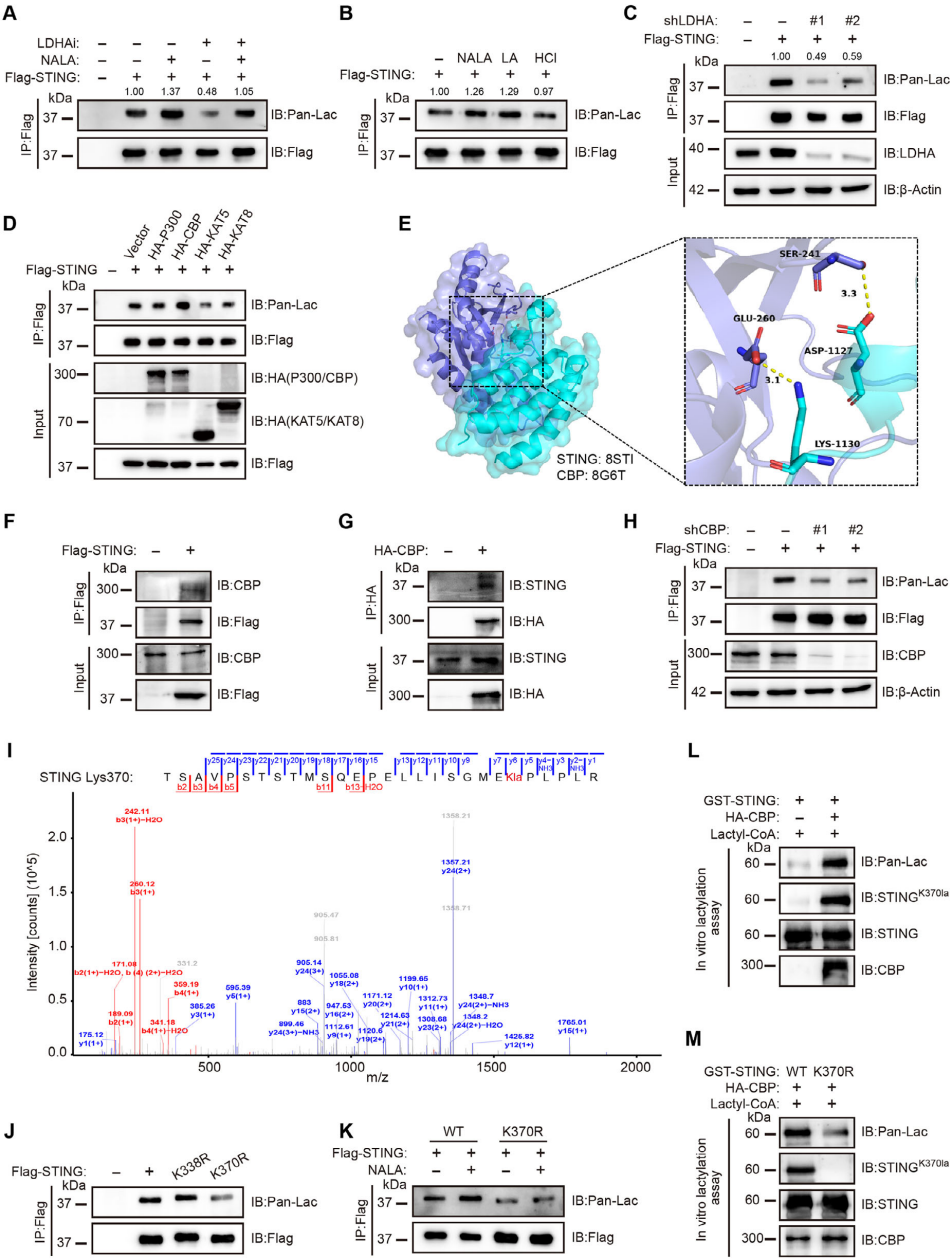
Identification of CBP‐mediated lactylation of STING at K370. (A) Co‐immunoprecipitation and western blot analysis of STING lactylation in HEK293T cells treated with NALA (25 mM) or LDHAi (15 mM) for 24 h. (B) Co‐immunoprecipitation and western blot analysis of STING lactylation in HEK293T cells treated with NALA (25 mM), lactic acid (LA, 25 mM), or HCl adjusted to the same pH as the LA treatment for 24 h. (C) Co‐immunoprecipitation and western blot analysis of STING lactylation in HEK293T cells following shRNA‐mediated knockdown of LDHA. (D) Screening of candidate lactyltransferases regulating STING lactylation by overexpression of the indicated enzymes, followed by immunoblot analysis. (E) Molecular docking analysis of human STING and CBP proteins. (F, G) Co‐immunoprecipitation and western blot analysis of the interaction between STING and CBP. (H) Co‐immunoprecipitation and western blot analysis of STING lactylation in HEK293T cells following shRNA‐mediated knockdown of CBP. (I) Representative mass spectrometry spectrum identifying K370 as a lactylation site on STING. (J) Co‐immunoprecipitation and western blot analysis of STING lactylation in HEK293T cells expressing Flag‐tagged STING‐WT, STING‐K338R, or STING‐K370R. (K) Co‐immunoprecipitation and western blot detection comparing lactylation levels of Flag‐tagged STING‐WT and STING‐K370R in HEK293T cells treated with or without NALA (25 mM) for 24 h before harvesting. (L) In vitro lactylation reaction using purified GST‐STING‐WT protein incubated with lactyl‐CoA in the presence or absence of purified HA‐CBP, followed by western blot analysis of STING lactylation. (M) In vitro lactylation reaction comparing purified GST‐STING‐WT and GST‐STING‐K370R proteins incubated with lactyl‐CoA and purified HA‐CBP, followed by western blot analysis of STING lactylation.

To examine whether STING undergoes lysine lactylation as a non‐histone protein, HEK293T cells were transfected with Flag‐tagged STING constructs. Cell lysates were then subjected to immunoprecipitation with anti‐Flag M2 agarose beads, followed by immunoblot analysis using a pan‐lactylation antibody. STING lactylation was readily detected under basal conditions. Treatment with NALA increased STING lactylation, whereas inhibition of lactate production by LDHAi reduced this modification (Figure 2A). Addition of exogenous lactate largely restored STING lactylation in LDHAi‐treated cells, suggesting that LDHA regulates STING lactylation mainly through the modulation of lactate availability. We next compared the effects of different treatments on STING lactylation. Both NALA and lactic acid increased STING lactylation, while HCl treatment did not produce a similar effect. These results indicate that lactate itself, rather than extracellular acidification, is responsible for the induction of STING lactylation (Figure 2B). Consistently, shRNA‐mediated knockdown of LDHA led to a pronounced decrease in STING lactylation levels (Figure 2C), supporting a functional link between lactate availability and STING lactylation. To identify the enzymatic machinery responsible for this modification, we screened candidate lactyltransferases and found that overexpression of CREB‐binding protein (CBP) increased STING lactylation levels (Figure 2D). Co‐immunoprecipitation assays and molecular docking analyses showed that CBP interacts with STING (Figure 2E–G), supporting a functional interaction between CBP and STING. Consistent with this observation, shRNA‐mediated knockdown of CBP resulted in a significant reduction in STING lactylation (Figure 2H). To map the specific lactylation sites on STING, mass spectrometry‐based proteomic analysis was performed, which identified lysine 338 (K338) and lysine 370 (K370) as candidate lactylation residues (Figure 2I; Figure S2A). To determine the functional relevance of these sites, Flag‐tagged STING mutants in which K338 or K370 was substituted with arginine (K338R or K370R) were generated and expressed in cells. Notably, mutation of K370, but not K338, substantially reduced the overall STING lactylation signal (Figure 2J,K). Although K338R did not diminish the pan‐lactylation signal, site‐specific analyses revealed an increase in K370 lactylation (Figure S2B). Moreover, only K370R, but not K338R, enhanced STING S366 phosphorylation (Figure S2C), identifying K370 as the predominant functional lactylation site. To determine whether CBP directly catalyzes STING lactylation, we conducted an in vitro lactylation assay with purified recombinant proteins. CBP markedly enhanced both pan‐lactylation and K370‐specific lactylation of STING‐WT in the presence of lactyl‐CoA, whereas mutation of K370 abolished the site‐specific lactylation signal (Figure 2L,M; Figure S2E), demonstrating that CBP directly catalyzes STING K370 lactylation. In addition, screening of candidate delactylases identified SIRT3 and SIRT4 as potential negative regulators of STING lactylation, since overexpression of these proteins markedly reduced STING lactylation levels (Figure S2D). Collectively, these findings demonstrate that STING is a bona fide lactylated non‐histone protein, with K370 serving as the major functional lactylation site and CBP acting as a direct regulator of STING K370 lactylation.

### 2.3 K370 Lactylation Suppresses STING Activation by Limiting TBK1 Recruitment and S366 Phosphorylation

**FIGURE 3:**
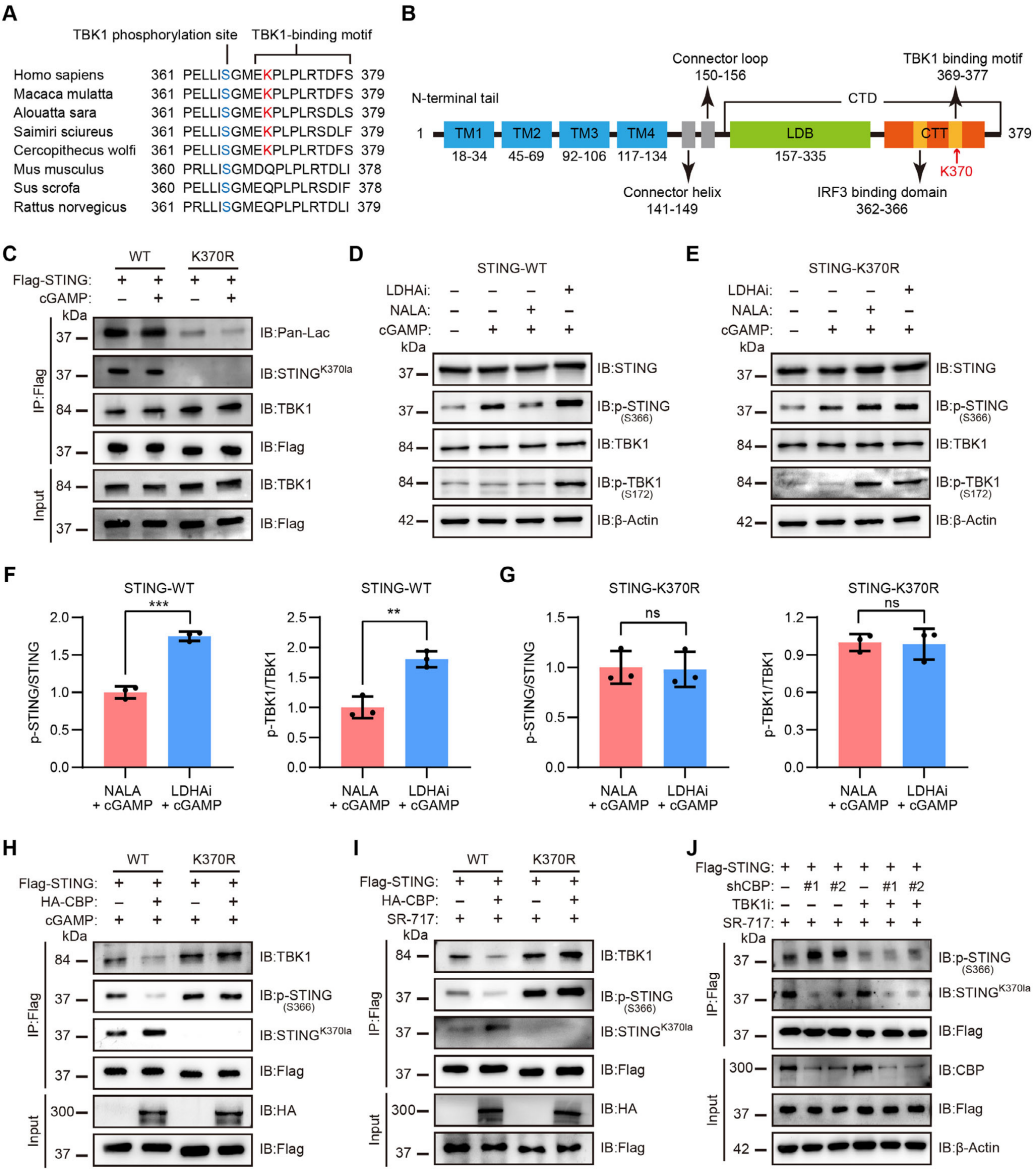
K370 lactylation impairs TBK1 recruitment and suppresses STING S366 phosphorylation. (A) Sequence alignment of the STING K370 region among the indicated species. (B) Schematic illustration of the domain architecture of STING. (C) Co‐immunoprecipitation and western blot detection of STING lactylation and STING‐TBK1 interaction in HEK293T cells expressing STING‐WT or STING‐K370R and stimulated with cGAMP (100 nM) for 6 h before harvesting. (D) Immunoblot evaluation of STING downstream signaling in cells expressing STING‐WT treated with NALA (25 mM) or LDHAi (15 mM) for 24 h, followed by cGAMP (100 nM) stimulation for 6 h. (E) Immunoblot evaluation of STING downstream signaling in cells expressing STING‐K370R treated with NALA (25 mM) or LDHAi (15 mM) for 24 h, followed by cGAMP (100 nM) stimulation for 6 h. (F, G) Quantification of the p‐STING/STING and p‐TBK1/TBK1 protein expression ratios in cells expressing STING‐WT or STING‐K370R following treatment with NALA (25 mM) or LDHAi (15 mM) for 24 h and subsequent stimulation with cGAMP (100 nM) for 6 h. Data are shown as mean ± SD (*n* = 3). *P*‐values by two‐tailed t‐test. ^**^ *p* < 0.01, ^***^ *p* < 0.001, ns: not significant. (H) Co‐immunoprecipitation and western blot detection of STING‐TBK1 interaction, STING K370 lactylation, and S366 phosphorylation in HEK293T cells expressing Flag‐tagged STING‐WT or STING‐K370R, with or without co‐transfection of HA‐CBP, followed by stimulation with cGAMP (100 nM) for 6 h before harvesting. (I) Co‐immunoprecipitation and western blot detection of STING‐TBK1 interaction, STING K370 lactylation, and S366 phosphorylation in U251 cells expressing Flag‐tagged STING‐WT or STING‐K370R, with or without co‐transfection of HA‐CBP, followed by stimulation with SR‐717 (10 µM) for 24 h before harvesting. (J) Co‐immunoprecipitation and western blot detection of STING K370 lactylation and STING S366 phosphorylation in U251 cells expressing Flag‐tagged STING‐WT following shRNA‐mediated knockdown of CBP, with or without treatment with the TBK1 inhibitor GSK8612 (5 µM), and stimulation with SR‐717 (10 µM) for 24 h before harvesting.

As shown in Figure 3B, human STING contains a short N‐terminal cytosolic region, four transmembrane helices, a cytosolic ligand‐binding domain (LBD), and a C‐terminal tail (CTT) [32]. The CTT contains binding motifs for IRF3 (residues 362–366) and TBK1 (residues 369–377), which are required for downstream signaling [32]. Sequence alignment among different species showed that lysine 370 (K370) is mainly conserved in primates, including humans and non‐human primates, but is not present in commonly used experimental animals such as mice, rats, and rabbits (Figure 3A). K370 is located within the TBK1‐binding region, suggesting that modification of this residue may affect the interaction between STING and TBK1. To examine this possibility, we carried out co‐immunoprecipitation assays in cells expressing Flag‐tagged STING following treatment with NALA or LDHAi. Inhibition of LDHA increased STING‐TBK1 binding, whereas lactate supplementation reduced the interaction (Figure S2F,G). Similar results were observed with the K370R mutant. Replacement of K370 with arginine, which prevents lactylation at this site, increased STING‐TBK1 association compared with wild‐type STING (Figure 3C). We next evaluated the effect of K370 lactylation on STING signaling. Stable cell lines expressing STING‐WT or STING‐K370R were generated. In STING‐WT cells, LDHA inhibition increased phosphorylation of STING and downstream signaling proteins (Figure 3D–F). In contrast, cells expressing STING‐K370R showed high levels of STING phosphorylation and downstream pathway activation following either NALA or LDHAi treatment (Figure 3E–G). These findings indicate that lactylation at K370 contributes to lactate‐mediated inhibition of STING signaling. Given that K370 resides within the TBK1‐binding motif, we next investigated how K370 lactylation influences TBK1 recruitment and subsequent S366 phosphorylation. Enhanced STING lactylation induced by CBP overexpression impaired STING‐TBK1 interaction and reduced S366 phosphorylation, whereas the K370R mutant retained stronger TBK1 binding and higher S366 phosphorylation (Figure 3H,I). CBP knockdown increased phosphorylation of STING at S366, whereas treatment with the TBK1 inhibitor markedly reduced this effect (Figure 3J; Figure S2H). Together, these data suggest that lactylation at K370 suppresses STING phosphorylation by reducing TBK1 association with STING.

### 2.4 K370 Lactylation of STING Modulates Its Ubiquitination States

**FIGURE 4:**
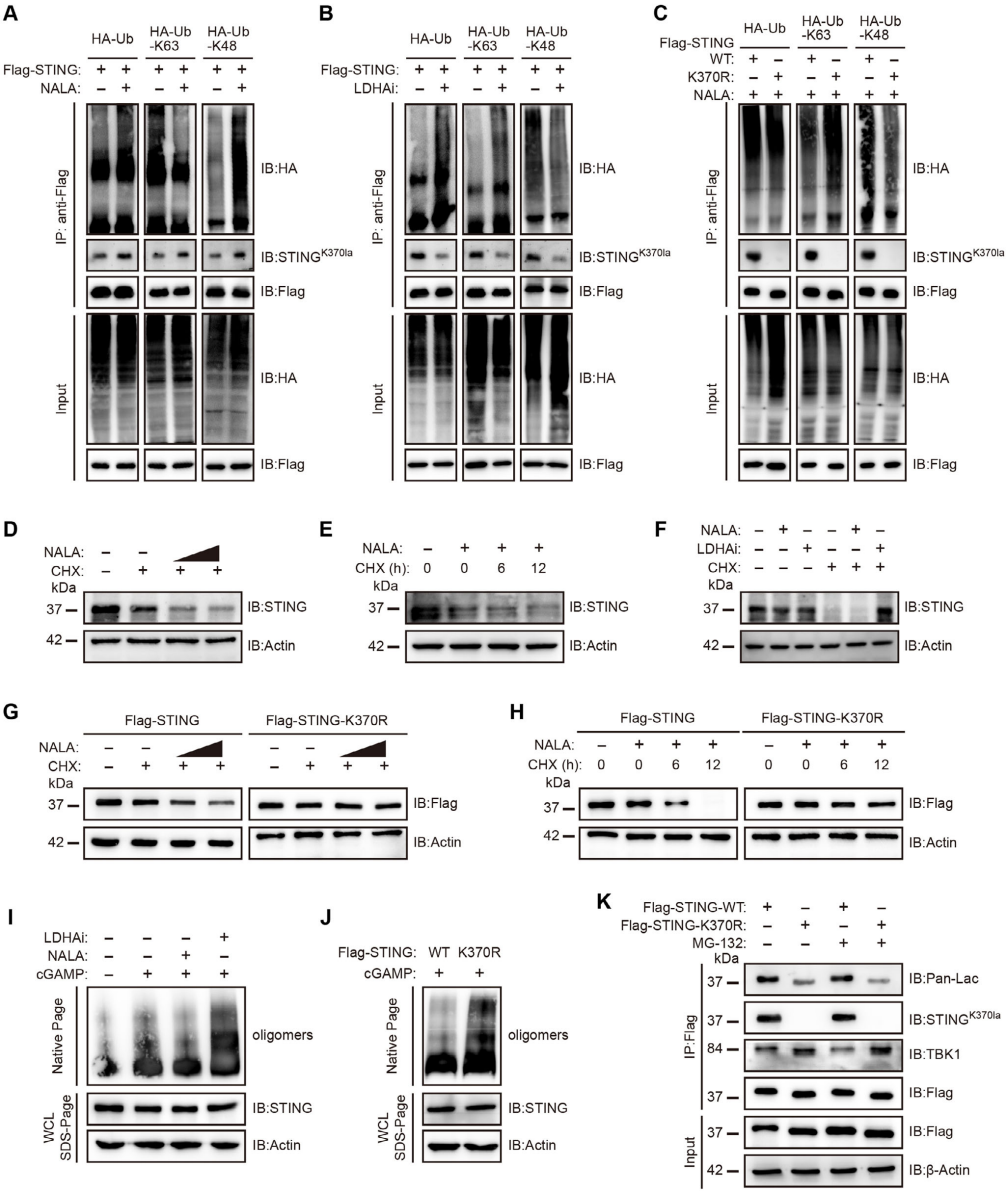
STING K370 lactylation rewires ubiquitin linkage and lowers protein stability and oligomerization. (A, B) HEK293T cells were transfected with Flag‐STING‐WT together with HA‐Ub, HA‐Ub‐K63, or HA‐Ub‐K48. After 24 h of transfection, cells were treated with NALA (25 mM) (A) or LDHAi (15 mM) (B) for 24 h, followed by MG132 (15 µM) treatment for 6 h before harvesting. (C) Co‐immunoprecipitation and western blot analysis of STING ubiquitination in HEK293T cells transfected with Flag‐STING‐WT or Flag‐STING‐K370R together with HA‐Ub, HA‐Ub‐K63, or HA‐Ub‐K48. After 24 h of transfection, cells were treated with NALA (25 mM) for 24 h, followed by MG132 (15 µM) treatment for 6 h before harvesting. (D) HEK293T cells were treated with increasing concentrations of NALA (15 and 25 mM) for 24 h, followed by CHX (100 ng/ml) treatment for 6 h before harvesting. Immunoblot analysis was performed to determine STING protein half‐life. (E) HEK293T cells were treated with NALA (25 mM) for 24 h, followed by CHX (100 ng/ml) treatment for the indicated times (6 and 12 h) before harvesting. Immunoblot analysis was performed to determine STING protein half‐life. (F) HEK293T cells treated with NALA (25 mM) or LDHAi (15 mM) for 24 h were subsequently treated with CHX (100 ng/ml) for 6 h before harvesting. Immunoblot analysis was performed to determine STING protein half‐life. (G) HEK293T cells transfected with Flag‐tagged STING‐WT or STING‐K370R were treated with increasing concentrations of NALA (15 and 25 mM) for 24 h, followed by CHX (100 ng/mL) treatment for 6 h before harvesting. Immunoblot analysis was performed to determine protein half‐life. (H) HEK293T cells transfected with Flag‐tagged STING‐WT or STING‐K370R were treated with NALA (25 mM) for 24 h, followed by CHX (100 ng/ml) treatment for the indicated times (6 and 12 h) before harvesting. Immunoblot analysis was performed to determine protein half‐life. (I) HEK293T cells transfected with Flag‐tagged STING‐WT were treated with NALA (25 mM) or LDHAi (15 mM) for 24 h and subsequently stimulated with cGAMP (100 nM) for 6 h before harvesting. Cell lysates were resolved by Native‐PAGE or SDS‐PAGE and subsequently analyzed by immunoblotting. (J) HEK293T cells transfected with Flag‐tagged STING‐WT or STING‐K370R were stimulated with cGAMP (100 nM) for 6 h before harvesting. Cell lysates were resolved by Native‐PAGE or SDS‐PAGE and subsequently analyzed by immunoblotting. (K) Co‐immunoprecipitation and western blot analysis of STING lactylation and STING‐TBK1 interaction in HEK293T cells expressing Flag‐tagged STING‐WT or STING‐K370R, with or without MG132 (15 µM) treatment for 6 h before harvesting.

Protein lactylation has been shown to regulate ubiquitin linkage patterns and protein stability in different biological settings [33, 34]. To examine whether K370 lactylation affects STING ubiquitination, we performed biochemical assays to analyze changes in STING ubiquitination. Lactate supplementation and LDHA inhibition caused changes in the overall ubiquitination pattern of STING (Figure S3A,B). We next analyzed the ubiquitin chains attached to STING using ubiquitin mutants that allow selective detection of K48‐ or K63‐linked polyubiquitination. Exogenous lactate supplementation increased K48‐linked ubiquitination and reduced K63‐linked ubiquitination of STING (Figure 4A). In contrast, LDHA inhibition promoted K63‐linked ubiquitination while diminishing K48‐linked ubiquitination of STING (Figure 4B), suggesting that lactate levels regulate the type of ubiquitin chains formed on STING. To determine whether these changes depend on K370 lactylation, we compared wild‐type STING with the lactylation‐defective STING‐K370R mutant. The K370R mutation decreased K48‐linked ubiquitination and increased K63‐linked ubiquitination, even in the presence of elevated lactate levels, suggesting that K370 lactylation contributes to the regulation of STING ubiquitination (Figure 4C). These results suggest that K370 lactylation promotes K48‐linked ubiquitination of STING while reducing K63‐linked ubiquitination, thereby affecting STING stability and signaling activity in response to metabolic changes.

### 2.5 K370 Lactylation Regulates STING Stability and Oligomerization

K48‐linked polyubiquitination is widely known to target proteins for proteasomal degradation and decrease their stability [35, 36, 37]. We next examined whether lactate affects STING stability by performing cycloheximide (CHX) chase assays to inhibit new protein synthesis. Increased lactate levels accelerated STING degradation, resulting in a shorter protein half‐life (Figure 4D,E). In contrast, LDHA inhibition slowed STING degradation and extended its half‐life (Figure 4F). We then investigated whether K370 lactylation contributes to lactate‐mediated regulation of STING stability. Compared with wild‐type STING, the lactylation‐deficient STING‐K370R mutant showed a longer half‐life under lactate‐rich conditions. These data indicate that K370 lactylation promotes STING turnover and contributes to the regulation of STING protein stability (Figure 4G,H). Beyond its role in protein stability, the pattern of STING ubiquitination also critically influences its higher‐order assembly and signaling competence. K63‐linked polyubiquitination has been implicated in promoting STING oligomerization, a prerequisite for efficient downstream signaling activation [38, 39, 40]. Upon pharmacological inhibition of lactate production, STING‐WT‐expressing cells exhibited a marked increase in STING oligomerization (Figure 4I). Consistently, STING‐K370R exhibited a higher degree of oligomerization than STING‐WT under comparable conditions (Figure 4J). Importantly, Similar results were observed in GBM cell lines, underscoring the robustness of this regulatory mechanism (Figure S3C–H).

To exclude the potential influence of proteasomal degradation on the assessment of STING lactylation, we examined STING lactylation in the presence of MG132. Proteasome inhibition had minimal effects on pan‐lactylation or K370‐specific lactylation of STING (Figure 4K; Figure S3I). Moreover, the enhanced STING‐TBK1 interaction and signaling observed in the K370R mutant remained evident following MG132 treatment (Figure 4K; Figures S3I–K and S2C). These findings indicate that K370 lactylation suppresses STING signaling not only by promoting proteasomal degradation but also by directly limiting STING‐TBK1 interaction independent of its effects on protein turnover.

### 2.6 Lactylation of STING at K370 Regulates Its ER‐to‐Golgi Trafficking

**FIGURE 5:**
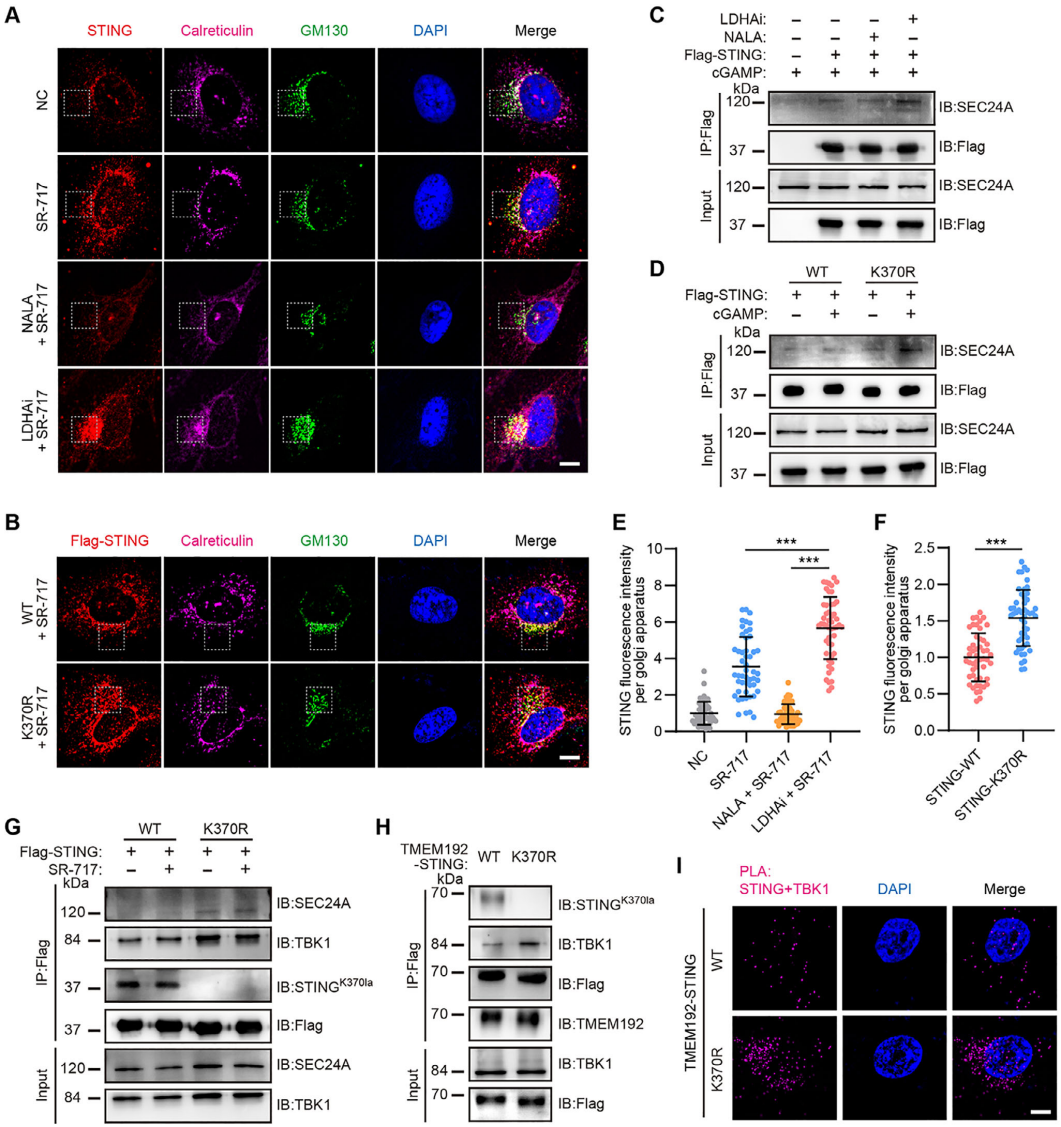
K370 lactylation attenuates COPII‐mediated ER‐to‐Golgi trafficking of STING. (A) Immunofluorescence analysis of the colocalization of endogenous STING with the endoplasmic reticulum (ER) and Golgi apparatus in U251 cells treated with NALA (25 mM) or LDHAi (15 mM) in the presence of SR‐717 (10 µM) for 24 h before harvesting. Scale bars: 10 µm. (B) Immunofluorescence analysis of the colocalization of Flag‐tagged STING‐WT or STING‐K370R with the ER and Golgi apparatus in U251 cells treated with SR‐717 (10 µM) for 24 h before harvesting. Scale bars: 10 µm. (C) Co‐immunoprecipitation and western blot analysis of the interaction between SEC24A and STING in HEK293T cells treated with NALA (25 mM) or LDHAi (15 mM) for 24 h and subsequently stimulated with cGAMP (100 nM) for 6 h before harvesting. (D) Co‐immunoprecipitation and western blot analysis of STING lactylation and its interaction with SEC24A in HEK293T cells expressing STING‐WT or STING‐K370R and stimulated with cGAMP (100 nM) for 6 h before harvesting. (E) Quantification of STING fluorescence intensity per Golgi apparatus. Data are shown as mean ± SD (*n* = 50). *P*‐values by one‐way ANOVA. ^***^ *p* < 0.001. (F) Quantification of STING fluorescence intensity per Golgi apparatus. Data are shown as mean ± SD (*n* = 50). *P*‐values by two‐tailed *t*‐test. ^***^ *p* < 0.001. (G) Co‐immunoprecipitation and western blot analysis of STING lactylation and its interactions with SEC24A or TBK1 in U251 cells treated with SR‐717 (10 µM) for 24 h before harvesting. (H) Co‐immunoprecipitation and western blot analysis of the interaction between Flag‐tagged TMEM192‐STING‐WT or TMEM192‐STING‐K370R and TBK1 in U251 cells. (I) Proximity ligation assay (PLA) analysis of the interaction between Flag‐tagged TMEM192‐STING‐WT or TMEM192‐STING‐K370R and TBK1 in U251 cells. Scale bar: 10 µm.

STING movement from the endoplasmic reticulum (ER) to the Golgi apparatus is required for efficient activation of downstream signaling, including STING phosphorylation and subsequent pathway activation [41, 42, 43]. We examined whether changes in lactate levels affect this trafficking process by analyzing STING distribution after lactate supplementation or LDHA inhibition. Immunofluorescence staining was used to detect the localization of STING together with the ER marker calreticulin and the Golgi marker GM130. Following STING agonist treatment, STING showed increased localization at the Golgi compared with the ER. However, addition of NALA prevented this redistribution and maintained STING retention in the ER, indicating that increased lactate levels restrict STING trafficking. LDHA inhibition produced the opposite effect and promoted STING accumulation at the Golgi (Figure 5A–E). Moreover, STING‐K370R displayed enhanced accumulation in the Golgi apparatus relative to the wild‐type protein (Figure 5B–F). Mechanistically, SEC24A, a key component of the COPII vesicle coat complex, has been reported to recognize the pSGME and pFS motifs within the C‐terminal region of STING (residues S366‐S379), thereby facilitating COPII‐mediated ER‐to‐Golgi export [44]. Co‐immunoprecipitation assays revealed that lactate accumulation weakened the interaction between STING and SEC24A, whereas inhibition of lactate production enhanced this association (Figure 5C). Furthermore, the STING‐K370R mutant exhibited increased association with SEC24A relative to the wild‐type protein, further supporting a role for K370 lactylation in modulating COPII engagement (Figure 5D–G; Figure S3J). Together, these findings show that K370 lactylation inhibits COPII‐dependent ER‐to‐Golgi trafficking of STING by reducing SEC24A binding, providing a mechanism through which lactate metabolism regulates STING activation.

### 2.7 STING Lactylation Inhibits TBK1 Binding Regardless of Subcellular Localization

STING trafficking from the ER to the Golgi is required for efficient downstream signaling, including activation of the STING‐TBK1 pathway and recruitment of downstream signaling proteins. We next examined whether the effect of K370 lactylation on STING‐TBK1 interaction was simply a consequence of altered ER‐to‐Golgi trafficking or whether K370 lactylation directly affected the interaction between these two proteins. According to the results shown in Figures 3C and 5G, the lactylation‐deficient STING‐K370R mutant showed increased association with TBK1 compared with wild‐type STING, both in the presence and absence of STING agonist stimulation. To distinguish whether K370 lactylation limits STING‐TBK1 signaling indirectly through impaired ER‐to‐Golgi trafficking or directly by weakening STING‐TBK1 interaction, we engineered an endolysosome‐localized TMEM192‐STING fusion protein and its corresponding K370R mutant. To re‐localize STING to endolysosomes, we substituted its four native transmembrane domains (which mediate ER retention) with the transmembrane domains derived from TMEM192, generating a TMEM192‐STING fusion protein that contains the STING C‐terminal region (amino acids 139–379) [45]. TMEM192 is a well‐characterized membrane protein localized to late endosomes and lysosomes [46]. Co‐immunoprecipitation analyses revealed that the TMEM192‐STING‐K370R mutant displayed a markedly enhanced association with TBK1 compared with the wild‐type fusion protein under basal conditions without exogenous STING agonist stimulation (Figure 5H; Figure S3K). To further substantiate this interaction within intact cells, we performed proximity ligation assays (PLA), which enable highly sensitive detection of protein‐protein interactions occurring within a 40 nm spatial range. Consistent with the biochemical data, PLA signals were significantly stronger in cells expressing TMEM192‐STING‐K370R than in those expressing the wild‐type construct, in the absence of agonist stimulation, indicating increased in situ engagement with TBK1 (Figure 5I). Taken together, these findings demonstrate that K370 lactylation can limit STING‐TBK1 engagement at a step that is separable from ER‐to‐Golgi trafficking.

### 2.8 STING K370 Lactylation is Clinically Associated With Poor Prognosis in GBM

**FIGURE 6:**
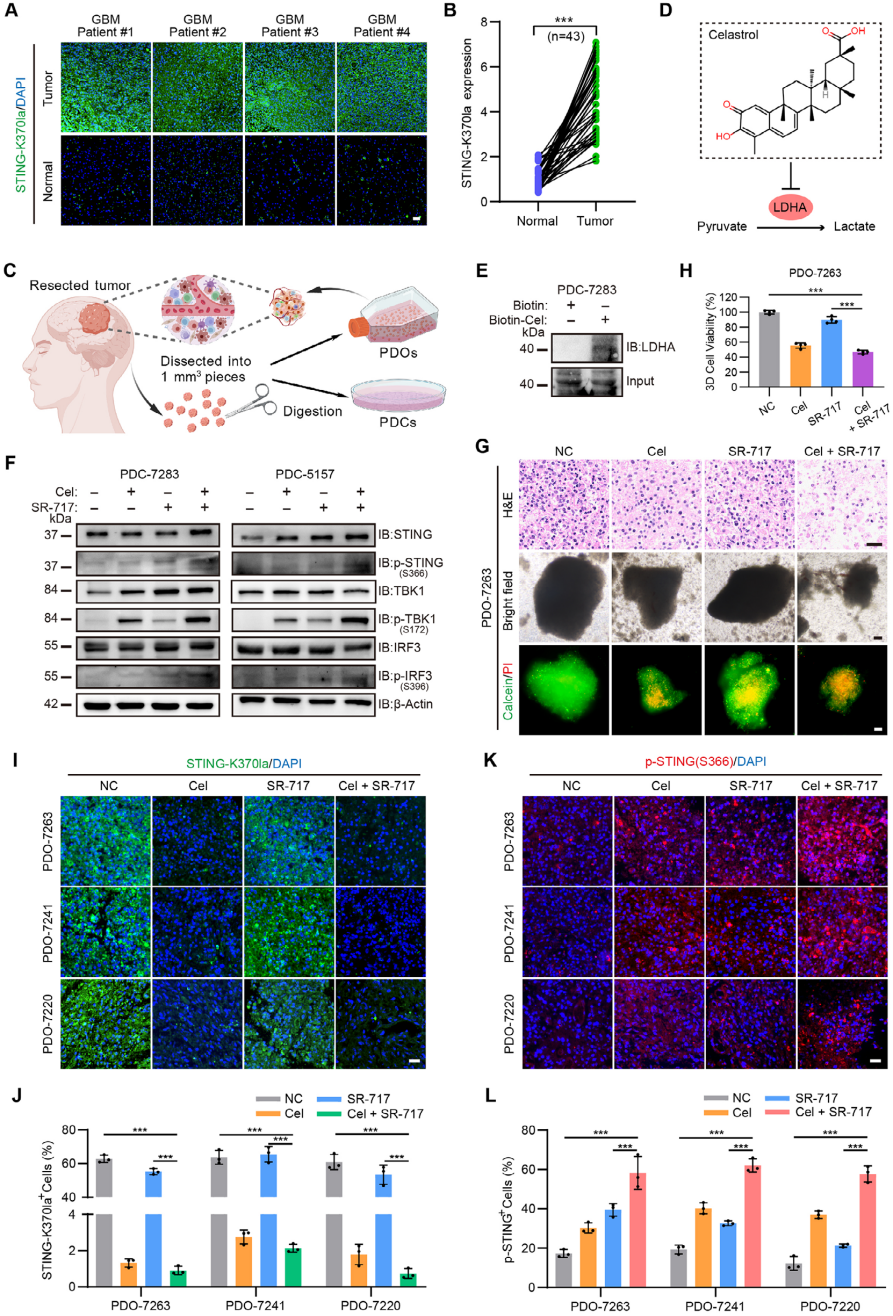
LDHA inhibition improves STING pathway activation and anti‐tumor response in patient‐derived models. (A) Representative immunofluorescence images of STING‐K370la in GBM tumor tissues and normal brain tissues. Scale bar: 50 µm. (B) Quantification of STING‐K370la immunofluorescence intensity in GBM tumor tissues and normal brain tissues. (*n* = 43). *P*‐values by two‐tailed *t*‐test. (C) Schematic illustration of the establishment and culture of patient‐derived GBM cells (PDCs) and patient‐derived GBM organoids (PDOs). This figure was created using BioGDP.com. (D) Schematic illustration of Celastrol‐mediated inhibition of LDHA. (E) Biotinylated‐protein pull‐down assay and western blot analysis evaluating the interaction between Celastrol (Cel) and LDHA in PDCs. (F) Western blot analysis of STING downstream signaling in PDCs treated with Cel (2 µM) and SR‐717 (5 µM) for 48 h. (G) Representative H&E staining, bright‐field morphology, and viability staining images of PDOs treated with Cel (2 µM) and SR‐717 (5 µM) for 72 h. Scale bars: 25, 100, 100 µm, top to bottom. (H) Three‐dimensional cell viability of PDOs treated with Cel (2 µM) and SR‐717 (5 µM) for 72 h, measured using the CellTiter‐Glo (CTG) luminescence assay. Data are shown as mean ± SD (*n* = 4). *P*‐values by one‐way ANOVA. ^***^ *p* < 0.001. (I) Representative immunofluorescence images of STING‐K370la (green) in PDOs subjected to the indicated treatments. Scale bars: 25 µm. (J) Quantification of STING‐K370la‐positive cells in PDOs following the indicated treatments. Data are shown as mean ± SD (*n* = 3). *P*‐values by one‐way ANOVA. ^***^ *p* < 0.001. (K) Representative immunofluorescence images of p‐STING (red) in PDOs subjected to the indicated treatments. Scale bars: 25 µm. (L) Quantification of p‐STING‐positive cells in PDOs following the indicated treatments. Data are shown as mean ± SD (*n* = 3). *P*‐values by one‐way ANOVA. ^***^ *p* < 0.001.

STING‐K370 lactylation (STING‐K370la) was detected in human GBM samples and showed relatively high levels in tumor tissues (Figure 6A,B). Survival analysis showed that patients with lower STING‐K370la levels had longer overall survival than patients with higher STING‐K370la levels (Figure S4A,B). These data suggest that increased STING‐K370la is associated with poor prognosis in GBM. To investigate the functional impact of lactate‐mediated STING regulation in a human‐relevant context, we established and cultured patient‐derived GBM cells (PDCs) and patient‐derived organoids (PDOs) (Figure 6C). These models were previously generated and extensively characterized in our prior studies [47, 48], and were confirmed to faithfully preserve the molecular genetic features of their corresponding primary tumors (Figure S4C). Notably, the PDOs additionally retained key histopathological characteristics of GBM, including vascular‐like structures and tumor architecture, thereby providing a physiologically relevant 3D platform for functional interrogation (Figure S4D,E). Cel, a bioactive triterpenoid compound with reported anti‐inflammatory and anti‐tumor activities [28], has been shown to target and suppress LDHA activity in sepsis [29, 49] (Figure 6D), and has also been reported to inhibit the growth of GBM tumor cells [30, 50]. To determine whether Cel directly interacts with LDHA in GBM cells, we generated biotin‐labeled Cel and performed pull‐down assays, which demonstrated a specific interaction between Cel and LDHA in PDCs (Figure 6E). Consistently, Cel treatment significantly reduced cell viability across multiple GBM cell lines (Figure S4F–H). Immunoblot analyses further showed that Cel treatment reduced LDHA abundance and globally decreased protein lactylation levels in GBM cells (Figure S4I–K). Importantly, Cel treatment markedly reduced K370 lactylation of STING in PDCs and concomitantly enhanced STING‐TBK1 interaction (Figure S5C).

### 2.9 Targeting LDHA Potentiates STING Activation and Suppresses Tumor Growth in Patient‐derived GBM Models

We next tested whether LDHA inhibition could enhance the response of GBM cells to STING activation by treating cells with Cel together with the STING agonist SR‐717. This combination increased the phosphorylation of STING, TBK1, and IRF3 compared with either treatment alone, indicating stronger activation of the STING signaling pathway (Figure 6F; Figure S5A,B). To further exclude potential compound‐specific effects of Cel, we performed both genetic and pharmacological validation in PDCs. Consistent with the effects of Cel, shRNA‐mediated knockdown of LDHA enhanced STING agonist‐induced STING S366 phosphorylation (Figure S5E). Moreover, two additional structurally distinct LDHA inhibitors, FX‐11 and Stiripentol (STP), similarly potentiated STING signaling and increased STING phosphorylation (Figure S5F,G). Notably, assessment of cytotoxicity in normal human astrocyte lines (SPGV12) revealed minimal toxicity at the concentrations used, suggesting limited cytotoxicity toward normal human astrocytes at the tested concentrations (Figure S5D).

Extending our analysis from 2D cell cultures to 3D organoid models, bright‐field images showed that PDOs in the combined treatment group of Cel and SR‐717 were considerably smaller than those in either monotherapy group or the untreated control group, with edge erosion observed (Figure 6G). H&E staining revealed that PDOs treated with Cel and SR‐717 exhibited loose structures and reduced cellular density (Figure 6G). Through Calcein/PI staining and the CellTiter‐Glo (CTG) assay, we observed a marked reduction in the 3D cell viability of PDOs following combined treatment with Cel and SR‐717 (Figure 6G,H). These findings were also confirmed in two additional PDO strains (Figure S6A–D). Further analysis of STING‐K370la, p‐STING (S366), and Ki67 expression was performed in PDOs derived from different GBM patients. The combined treatment resulted in a marked decrease in Ki67 positivity across all three PDO lines, consistent with suppressed proliferative activity (Figure S6E,F). Consistent with the proposed mechanism, STING‐K370la levels were significantly reduced across all PDO lines following combination treatment, accompanied by a substantial increase in p‐STING (S366) positive cells (Figure 6I–L). Given the observed increase in STING S366 phosphorylation, we next examined the downstream induction of type I interferon (IFN) signaling [51]. Consistent with this paradigm, immunofluorescence analyses performed in both PDCs and PDOs demonstrated that combined treatment with Cel and the STING agonist SR‐717 markedly increased IFN‐β expression compared with either agent alone (Figures S5H,I and S6G,H). Together, these findings demonstrate that LDHA inhibition enhances STING agonist‐induced activation of the STING‐TBK1‐IFN axis in patient‐derived GBM models.

### 2.10 LDHA Inhibition Enhances STING‐driven Immune Remodeling and Potentiates PD‐1 Blockade in GBM PDOs

**FIGURE 7:**
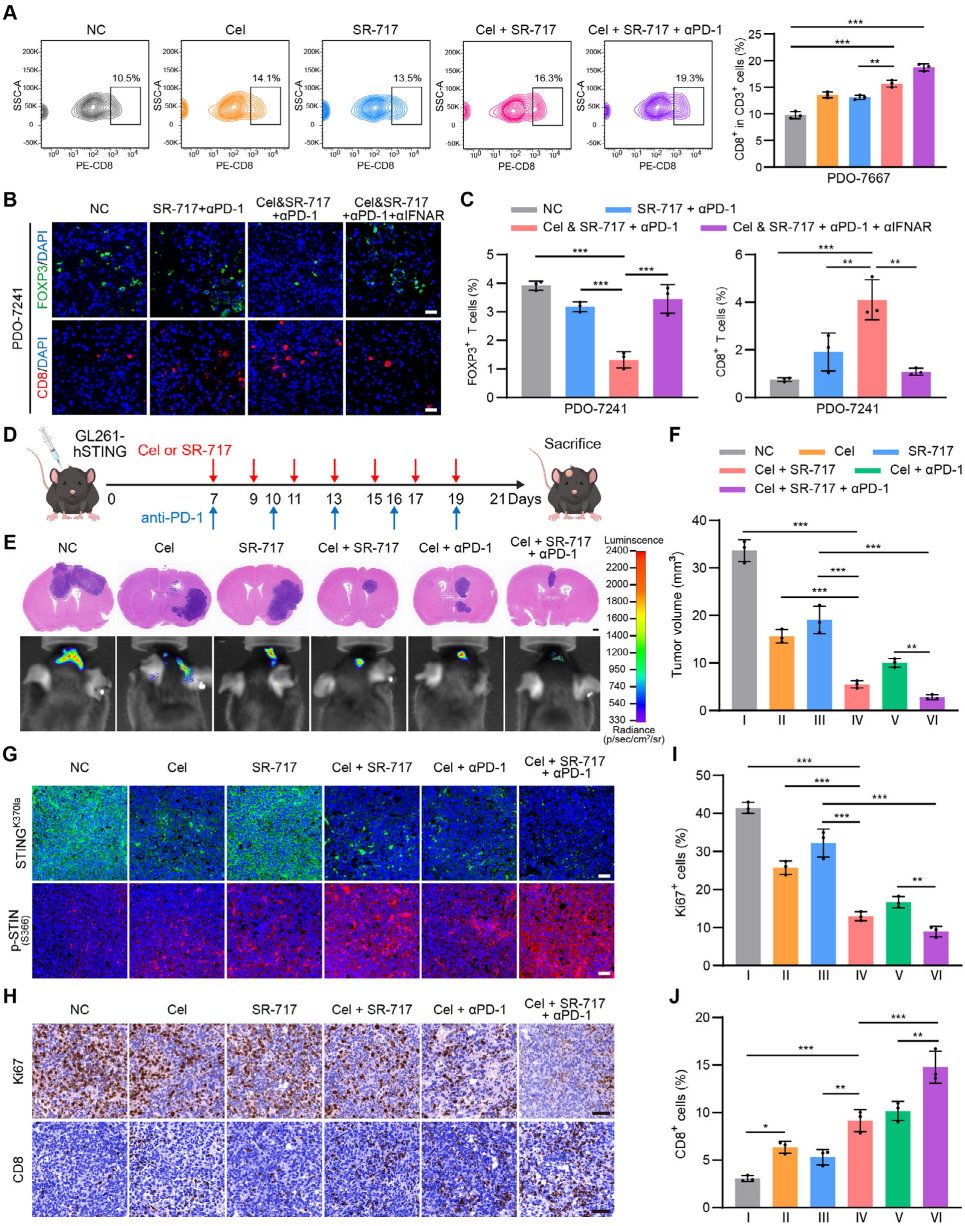
LDHA inhibition potentiates the therapeutic efficacy of STING activation and PD‐1 blockade in GBM. (A) Flow cytometric analysis of CD45^+^CD3^+^CD8^+^ T cells in PDOs cultured for less than 2 weeks and treated under the indicated regimens, including Cel (2 µM), SR‐717 (5 µM), and anti‐PD‐1 antibody, for 5 days. Data are shown as mean ± SD (*n* = 3). *P*‐values by one‐way ANOVA. ^**^ *p* < 0.01, ^***^ *p* < 0.001. (B) Representative immunofluorescence visualization of FOXP3 and CD8 in PDOs subjected to the indicated treatment regimens, including Cel (2 µM), SR‐717 (5 µM), anti‐PD‐1 antibody, and anti‐IFNAR antibody, for 5 days. Scale bars: 25 µm. (C) Statistical analysis of FOXP3^+^ and CD8^+^ cells in PDOs following the indicated treatment regimens. Data are shown as mean ± SD (*n* = 3). *P*‐values by one‐way ANOVA. ^**^ *p* < 0.01, ^***^ *p* < 0.001. (D) Schematic illustration of the orthotopic GL261‐hSTING glioblastoma (GBM) model and treatment schedule. Mice received Cel (2 mg /kg), SR‐717 (15 mg/kg), and/or anti‐PD‐1 antibody (10 mg/kg) according to the indicated regimens. This figure was created using BioGDP.com. (E) Representative H&E staining images of coronal brain sections and representative bioluminescence imaging of tumor‐bearing mice at the experimental endpoint. For bioluminescence imaging, mice were injected with D‐luciferin (150 mg/kg) prior to image acquisition. Scale bar: 500 µm. (F) Quantitative assessment of intracranial tumor volumes. Data are shown as mean ± SD. *P*‐values by one‐way ANOVA. ^**^ *p* < 0.01, ^***^ *p* < 0.001. (G) Immunofluorescence detection of STING‐K370la and p‐STING (S366) in tumor tissues from the indicated treatment groups. Scale bars: 50 µm. (H) Immunohistochemical detection of Ki67 and CD8 in tumor tissues from the indicated treatment groups. Scale bars: 50 µm. (I, J) Quantitative evaluation of Ki67‐positive and CD8‐positive cell percentages in tumor tissues from the indicated treatment groups. Data are shown as mean ± SD (*n* = 3). *P*‐values by one‐way ANOVA. ^*^ *p* < 0.05, ^**^ *p* < 0.01, ^***^ *p* < 0.001. Groups I‐VI correspond to NC, Cel, SR‐717, Cel + SR‐717, Cel + αPD‐1, and Cel + SR‐717 + αPD‐1, respectively.

Importantly, the established PDOs preserved key components of the immune microenvironment present in the original tumor tissues, including tumor‐associated macrophages (TAMs) and lymphocyte populations (Figure S7A,B). Consistent with the highly immunosuppressive nature of GBM, untreated PDOs exhibited a microenvironment enriched in M2‐like TAMs and FOXP3^+^ regulatory T (Treg) cells, closely recapitulating the immune landscape of the corresponding patient tumors. To investigate whether metabolic intervention and STING activation could alleviate the immunosuppressive microenvironment of GBM, we first analyzed T‐cell populations in PDOs by flow cytometry. Both Cel and SR‐717 monotherapies increased the proportion of CD8^+^ T cells within the CD3^+^ T‐cell compartment, while their combination produced a further increase. Notably, the addition of anti‐PD‐1 antibody resulted in the highest proportion of CD8^+^ T cells among CD3^+^ T cells (Figure 7A; Figure S9A). These results indicate that LDHA inhibition enhances STING agonist‐driven expansion of CD8^+^ T‐cell populations and further potentiates the immunomodulatory effects of PD‐1 blockade within the preserved PDO immune microenvironment. We next evaluated the effects of combined Cel, SR‐717, and anti‐PD‐1 treatment in GBM PDO models. Compared with SR‐717 plus anti‐PD‐1 treatment, the addition of Cel further inhibited organoid growth and decreased 3D cell viability (Figure S7C–E and I–K). This treatment also increased IFN‐β expression (Figure S7F–H), suggesting enhanced activation of STING‐dependent innate immune signaling. These results show that LDHA inhibition improves the therapeutic response to STING activation combined with PD‐1 blockade in GBM PDOs. We then examined whether the increased IFN‐β response was accompanied by changes in T‐cell composition within PDOs. Immunofluorescence analysis showed that triple therapy increased the proportion of CD8^+^ T cells and reduced FOXP3^+^ Treg cells compared with SR‐717 plus anti‐PD‐1 treatment (Figure 7B,C), consistent with the flow cytometry results. To determine the role of type I interferon signaling in these changes, PDOs were treated with an IFN‐α/β receptor (IFNAR)‐blocking antibody. IFNAR inhibition reduced the increase in CD8^+^ T cells and restored FOXP3^+^ Treg cell accumulation induced by the triple combination treatment (Figure 7B,C). These findings identify type I interferon signaling as a critical mediator linking STING activation to T‐cell–oriented immune remodeling within the GBM microenvironment. Further analysis showed that the combination treatment also altered tumor‐associated macrophage (TAM) populations in PDOs. The proportion of CD68^+^CD206^+^ M2‐like TAMs was decreased, whereas CD68^+^CD86^+^ M1‐like TAMs were increased after combination therapy. Notably, these effects were largely attenuated by IFNAR blockade (Figure S7L,O). Furthermore, the immune‐modulatory effects of the triple‐combination regimen were independently validated in additional patient‐derived PDO models, where increased CD8^+^ T‐cell abundance, reduced FOXP3^+^ Treg‐cell frequencies, and similar shifts in TAM phenotypes were consistently observed (Figure S8A–H). Collectively, these findings establish type I interferons downstream of STING‐TBK1 signaling as key mediators of immune microenvironment remodeling induced by LDHA inhibition and STING activation, providing mechanistic insight into how metabolic intervention enhances the efficacy of immunotherapy in GBM.

### 2.11 LDHA Inhibition Enhances STING Activation and Antitumor Immunity in an Orthototic GBM Model

To determine whether the antitumor efficacy and immune activation observed in patient‐derived models could also be observed in vivo, we established an orthotopic GL261‐hSTING glioblastoma model and evaluated the therapeutic efficacy of Cel, SR‐717, and anti‐PD‐1 treatment (Figure 7D). Western blot analysis confirmed stable expression of human STING in GL261‐hSTING cells and demonstrated that Cel enhanced SR‐717‐induced activation of the STING signaling pathway in vitro (Figure S9B,C). Consistent with the findings obtained in PDO models, triple‐combination treatments exerted significant antitumor activity in vivo. Histological examination of coronal brain sections and bioluminescence imaging revealed a marked reduction in intracranial tumor burden in mice receiving the triple‐combination treatments compared with control‐treated animals (Figure 7E). Quantitative analysis further demonstrated that combined treatment with Cel and SR‐717 significantly reduced tumor volume, whereas the addition of anti‐PD‐1 antibody produced the most pronounced tumor‐suppressive effect among all treatment groups (Figure 7F).

To investigate the underlying mechanism, we examined STING lactylation and pathway activation in tumor tissues. Immunofluorescence analyses showed that Cel‐containing treatment regimens markedly reduced STING‐K370 lactylation, accompanied by enhanced phosphorylation of STING at Ser366 (Figure 7G). These findings support a model in which LDHA inhibition relieves lactylation‐mediated suppression of STING signaling. This mechanism is consistent with the enhanced STING activation observed in vivo. We further examined tumor cell proliferation and immune cell infiltration after treatment. Immunohistochemical staining showed a gradual decrease in Ki67‐positive tumor cells among the treatment groups, with the lowest level detected in mice treated with the triple combination (Figure 7H,I). In contrast, the number of CD8^+^ T cells increased after treatment, with the highest infiltration observed in the Cel plus SR‐717 plus anti‐PD‐1 group (Figure 7H,J). These findings suggest that LDHA inhibition enhances the effects of STING activation and PD‐1 blockade by reducing tumor cell growth and promoting antitumor immune responses.

## 3 Discussion

**FIGURE 8:**
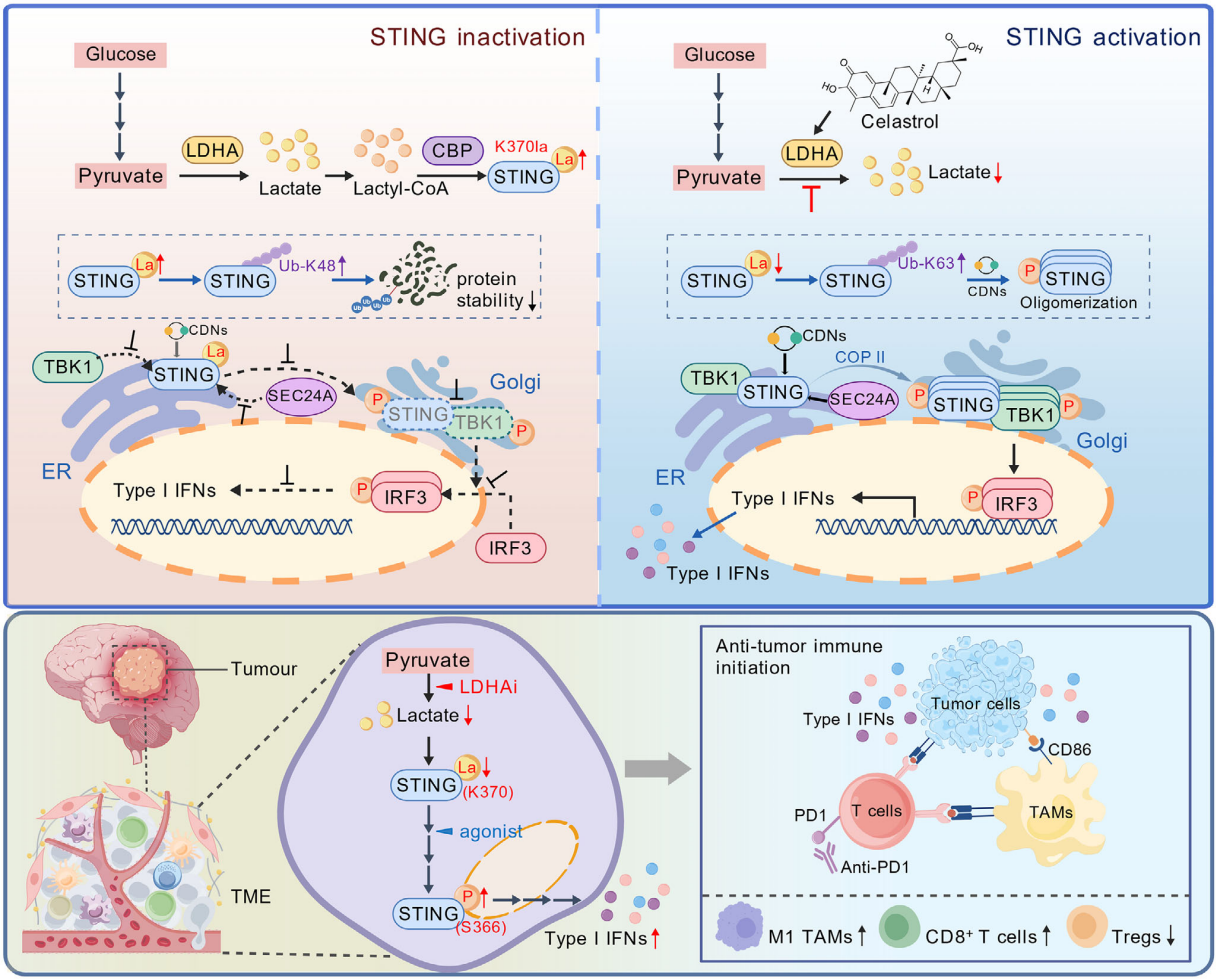
Schematic model illustrating lactate‐driven STING K370 lactylation as a metabolic checkpoint restraining STING‐TBK1 signaling and downstream anti‐tumor immune responses. Under conditions of lactate accumulation, STING undergoes site‐specific lactylation at the primate‐conserved lysine 370 (K370) residue within the TBK1‐binding motif. K370 lactylation directly weakens STING‐TBK1 interaction and biases STING ubiquitination toward K48‐linked chains while suppressing K63‐linked ubiquitination, resulting in reduced protein stability and impaired oligomerization. In parallel, K370 lactylation attenuates the interaction between STING and the COPII component SEC24A, thereby limiting ER‐to‐Golgi trafficking, a process required for efficient amplification of STING‐TBK1‐IRF3 signaling. Conversely, suppression of lactate production diminishes STING K370 lactylation, restores STING‐TBK1 association, favors K63‐linked ubiquitination, stabilizes STING, and permits efficient ER‐to‐Golgi translocation, thereby enabling robust activation of type I interferon signaling and innate immune responses. Activated STING signaling induces type I interferon responses, thereby fostering anti‐tumor immunity and promoting a more immunostimulatory tumor microenvironment. This figure was created using BioGDP.com.

Our study identifies K370 lactylation as an important regulator of STING signaling. This modification reduces STING‐TBK1 interaction and suppresses the initial activation of the STING pathway. In addition, K370 lactylation changes the ubiquitination pattern of STING by increasing K48‐linked ubiquitination and reducing K63‐linked ubiquitination, resulting in decreased STING stability and impaired oligomerization. K370 lactylation also affects ER‐to‐Golgi trafficking of STING by reducing its interaction with SEC24A, which is required for efficient activation of downstream STING‐TBK1‐IRF3 signaling. Reconstitution experiments further show that K370 lactylation directly inhibits STING‐TBK1 binding independent of changes in STING localization. Together, these results demonstrate that K370 lactylation regulates STING activity through multiple mechanisms, including control of protein stability, trafficking, and signaling complex formation, linking cellular metabolic status to innate immune suppression (Figure 8).

Previous studies have shown that K48‐linked ubiquitination promotes STING degradation through the proteasome pathway [52, 53], whereas K63‐linked ubiquitination facilitates STING oligomerization and downstream signaling activation [38, 39, 40]. For example, DAPK3 was reported to regulate STING ubiquitination by reducing K48‐linked ubiquitination while increasing K63‐linked modification, thereby enhancing anti‐tumor immune responses [15]. In this study, we identify lactate‐induced STING lactylation as an upstream regulatory event that connects metabolic changes with STING protein regulation. Increased lactate availability promotes K370 lactylation, which shifts STING ubiquitination toward the degradative K48‐linked form and decreases STING stability. In contrast, reduced lactate levels decrease STING lactylation, favor K63‐linked ubiquitination, and promote STING activation. This lactate‐dependent regulatory mechanism provides an additional metabolic layer controlling STING signaling and may contribute to immune suppression in tumors with high lactate production.

Our mechanistic studies were performed using human cellular models and proteins, revealing a regulatory mechanism that is not conserved in commonly used rodent systems. The K370 residue is conserved in primates but absent in rodents, indicating species‐specific differences in STING regulation. One possible explanation is that the K370 site may have evolved in primates as an additional regulatory element controlling STING activity, whereas this mechanism is absent in rodents. Such differences are consistent with previous studies showing distinct regulatory patterns and agonist responses between human and mouse STING proteins [13]. These findings highlight the importance of combining animal models with human‐based experimental systems when studying STING biology and developing STING‐targeted therapies [14, 15].

To examine the biological significance and therapeutic relevance of this regulatory mechanism, we used both patient‐derived tumor organoids (PDOs) and an orthotopic glioblastoma mouse model. PDOs maintain important features of primary tumors, including tissue organization, cellular diversity, and immune‐related components [54, 55]. Compared with conventional organoids generated from dissociated tumor cells, the PDOs established in our previous studies preserve native cell–cell interactions and microenvironmental characteristics [56, 57], providing a useful model for studying human tumor biology and metabolic regulation [54, 56, 58]. In addition, the orthotopic glioblastoma model allowed us to evaluate therapeutic effects in vivo within an intact immune environment. The combination of these complementary models supports the relevance of the lactate‐STING regulatory axis and provides further evidence for its potential as a therapeutic target.

In summary, our study identifies lactate‐induced STING lactylation as a metabolic regulatory mechanism that connects tumor lactate accumulation with innate immune suppression in human cancers. These findings provide new insights into how increased lactate production may promote resistance to immunotherapy and highlight the need to consider both animal models and human‐based systems when studying primate‐specific immunometabolic regulation. Therapeutically, we demonstrate that pharmacological LDHA inhibition with Cel reduces STING lactylation, restores STING signaling competence, and synergizes with STING agonism and anti‐PD‐1 therapy across patient‐derived GBM models and orthotopic glioblastoma mouse models. More broadly, our work nominates STING‐K370 lactylation as a candidate biomarker associated with poor prognosis in GBM, warranting future validation in independent cohorts, and identifies it as a potential target for combinatorial immunotherapy in lactate‐rich malignancies. Together, these insights advance our understanding of how tumor metabolism shapes anti‐tumor immunity and provide a clinically relevant rationale for integrating metabolic and immune intervention in GBM.

## 4 Methods

### 4.1 Cell Lines and Clinical Samples

Cell lines U251, SVGp12, HEK293T, and GL261 were procured from American Type Culture Collection (ATCC) and maintained in DMEM supplemented with 10% FBS, 100 U/mL penicillin & streptomycin in a 5% CO_2_ atmosphere at 37°C. PDC‐7283, PDC‐5157 were sourced from Shanghai East Hospital and cultured in DMEM/F12 supplemented with 10% FBS, 100 U/mL penicillin & streptomycin.

All human tissue samples were obtained through standard clinical procedures with ethical approval from the Ethical Committee of Shanghai East Hospital, School of Medicine, Tongji University (Approval number: 2025294) following informed consent. Both tumor and matched normal adjacent tissues were collected during surgical resection, with their identities pathologically confirmed. Detailed sample information corresponding to each identification number is provided in Table S1.

### 4.2 Orthotopic Glioblastoma Mouse Model and in Vivo Treatment

All animal procedures were conducted in accordance with the National Institutes of Health Guide for the Care and Use of Laboratory Animals and were approved by the Animal Care Committee of Tongji University (Approval No. TJAA11026102). Six‐week‐old female C57BL/6 mice were anesthetized and stereotactically inoculated with 1 × 10^5^ GL261‐hSTING cells into the cerebral hemisphere at a depth of 3.0 mm. The GL261‐hSTING line was generated by stable expression of human STING in GL261 cells. Following tumor establishment, animals were randomly allocated to six experimental groups, with five mice in each group. Celastrol (2 mg/kg) and SR‐717 (15 mg/kg) were administered by intraperitoneal injection on alternate days, whereas anti‐PD‐1 antibody (10 mg/kg) was administered intraperitoneally every 3 days. Treatments were maintained for 14 consecutive days according to the designated regimen. At study completion, mice were euthanized, and brain tissues were collected for subsequent histopathological and immunostaining analyses. Tumor volume was determined using the formula *V* = *ab* ^2^/2 (*a* > *b*), where *a* and *b* denote the longest and shortest tumor diameters, respectively.

### 4.3 Isolation and Culture of Patient‐derived Glioblastoma Cells

Patient‐derived glioblastoma cells (PDCs) were generated following procedures described in our previously published studies [47, 48]. Tumor tissues were placed in DMEM/F12 and transported on ice within 30 min. Tissues were washed with cold PBS containing antibiotics, cut into small fragments, and enzymatically dissociated with collagenase IV at 37°C. The digested samples were filtered through a cell strainer to obtain single‐cell suspensions. After red blood cell removal, cells were collected by centrifugation and resuspended for subsequent culture.

### 4.4 Construction of Patient‐derived Glioblastoma Organoids

Patient‐derived glioblastoma organoids (PDOs) were generated according to methods reported in our previously published studies [47, 48]. Briefly, fresh GBM patient specimens were soaked in washing solution for three rounds and cut into approximately 0.5–1 mm^3^ microtissue blocks. Then the microtissue blocks were collected and transferred to a new dish with abundant culture medium. The PDOs were maintained in the culture medium 25% DMEM, 25% DMEM‐F12, supplemented with 1 × PenStrep, 50% Neurobasal, 1 × N2 supplement, 1 × GlutaMax, 1 × B27 w/o vitamin A supplement, 1 × NEAAs, 1 × 2‐mercaptoethanol, 20 ng/mL FGF, and 20 ng/mL EGF.

### 4.5 Immunofluorescence Staining

HEK293T cells, U251 cells, and PDCs were seeded on coverslips and stimulated as indicated. Cells were fixed by 4% paraformaldehyde, permeabilized via 0.1% Triton X‐100, and subsequently blocked in 1× PBS containing 3% BSA for 1 h. Normal brain tissue samples, GBM tumor tissue samples, and PDOs were fixed overnight in 4% paraformaldehyde. Paraffin‐fixed samples were sectioned into pieces, dewaxed, and hydrated. The sections were blocked and incubated with primary antibodies (1:100) overnight at 4°C. Secondary antibodies (1:200) were used at room‐temperature for 1 h. Mounted samples were imaged by a high‐resolution confocal microscope (Nikon, NSPARC, Japan). Details of the primary antibodies are provided in Table S2.

### 4.6 Mass Spectrometry Analysis

To identify STING lactylation sites, Flag‐tagged STING was immunopurified from NALA‐treated HEK293T cells and separated by SDS‐PAGE. The corresponding Flag‐STING band was excised and analyzed by LC–MS/MS using an Orbitrap Exploris 480 mass spectrometer. Peptides were separated by nano‐liquid chromatography and subjected to tandem mass spectrometry analysis for lactylation site identification.

### 4.7 Co‐Immunoprecipitation and Western Blotting

For co‐immunoprecipitation assays, cells expressing the indicated tagged proteins or endogenous proteins were lysed and subjected to immunoprecipitation using the corresponding antibodies or affinity resins. The precipitated complexes were analyzed by SDS‐PAGE followed by immunoblotting. Detailed information on antibodies used in this study is provided in Table S2.

### 4.8 In Vitro Lactylation Assay

Purified GST‐STING proteins were incubated with HA‐CBP purified from HEK293T cells in lactylation buffer containing 50 mM HEPES (pH 7.8), 30 mM KCl, 0.25 mM EDTA, 5 mM MgCl_2_, 5 mM sodium butyrate, 2.5 mM DTT, and 20 mM lactyl‐CoA. Reactions were carried out at 30°C for 30 min and terminated by addition of SDS sample buffer. Samples were heated at 100°C for 5 min, separated by SDS‐PAGE, and analyzed by immunoblotting with the indicated antibodies.

### 4.9 Native Polyacrylamide Gel Electrophoresis

Cells were lysed in NP‐40 buffer, and protein concentrations were determined using a BCA assay. Equal amounts of protein were prepared in native loading buffer and separated by Blue Native PAGE using the Native PAGE system (Beyotime, P0543S). Electrophoresis was performed at 150 V under cold conditions. Proteins were then analyzed by immunoblotting.

### 4.10 Proximity Ligation Assay (PLA)

Cells were fixed with 4% paraformaldehyde and permeabilized with 0.5% Triton X‐100 in methanol. After RNase A treatment, protein–protein interactions were detected using the Duolink PLA kit (Sigma–Aldrich). Cells were incubated with primary antibodies, followed by PLA probe hybridization, ligation, and signal amplification according to the manufacturer's instructions. Nuclei were stained with DAPI, and images were acquired using a Nikon Eclipse Ti2‐E microscope with a 60× objective.

### 4.11 Pull‐Down of Celastrol‐Bind Proteins

Celastrol was labeled with biotin using a biotinylation reagent kit and purified by desalting. Cell lysates were incubated with biotinylated Celastrol or biotin control at 4°C overnight. The complexes were collected using streptavidin agarose beads (Beyotime, P0654S). After washing, bound proteins were eluted and analyzed by western blotting to detect LDHA binding.

### 4.12 Cell Viability Assay

For CCK‐8 assays, PDCs and U251 cells were seeded in 96‐well plates at 5 × 10^3^ cells per well and allowed to attach for 24 h. Cells were then treated as indicated and cultured for an additional 48 h. Cell viability was measured using a CCK‐8 kit (KeyGEN, KGA9305).

For 3D viability analysis, PDOs were cultured in 48‐well plates and treated as indicated after 24 h of recovery. Cell viability was assessed after 72 or 120 h using the CellTiter‐Glo 3D assay kit (KeyGEN, KGA9309). Absorbance and luminescence signals were recorded using a Spark microplate reader (TECAN).

For live/dead staining, PDOs were incubated with Calcein AM and propidium iodide (PI) using a viability/cytotoxicity assay kit (Beyotime, C2015). Following a 30 min incubation at 37°C in the dark, fluorescence images were acquired using a Nikon Ti2‐E inverted fluorescence microscope.

### 4.13 Flow Cytometry Analysis

Flow cytometric analyses were performed using PDOs cultured for less than 2 weeks. Organoids were harvested, washed with PBS, and dissociated into single cells using Accutase (Gibco). Digestion was terminated with FACS buffer (PBS supplemented with 2% FBS). Cells were collected by centrifugation, resuspended in FACS buffer, and filtered through a 70 µm cell strainer. Following cell counting, Fc receptors were blocked with Human TruStain FcX (BioLegend, 422302) for 10 min. Cells were then stained with fluorophore‐conjugated antibodies for 20 min at room‐temperature in the dark. After washing, samples were analyzed by flow cytometry. Antibody information is provided in Table S2.

### 4.14 H&E Staining and Immunohistochemistry (IHC)

Human brain tissues, GBM specimens, PDOs, and mouse brain tissues were fixed in 4% paraformaldehyde and embedded in paraffin. Paraffin sections were prepared for subsequent staining. For H&E staining, sections were deparaffinized, rehydrated, and stained with hematoxylin and eosin. For IHC analysis, tissue sections underwent antigen retrieval and blocking before incubation with primary antibodies and secondary antibodies. Immunoreactive signals were visualized using DAB substrate, and images were collected with a Nikon Ti2‐E microscope.

### 4.15 Statistical Analysis

Statistical analyses were performed using GraphPad Prism 8 (GraphPad Software). Data are presented as mean ± SD from independent experiments. Comparisons between two groups were conducted using unpaired two‐tailed Student's *t*‐tests, whereas comparisons among multiple groups were analyzed by one‐way ANOVA followed by Tukey's multiple‐comparison test. Statistical significance was defined as ^*^ *p* < 0.05, ^**^ *p* < 0.01, ^***^ *p* < 0.001, and ^****^ *p* < 0.0001.

## Author Contributions

J.Z. and C.Z. designed and led the project. Y.W., J.W., X.C., and W.P. performed experiments. Y.Y., H.X., H.W., and C.Z. analyzed data. S.X. and Q.W. provided clinical samples and information. J.Z. and C.Z. guided data analysis. J.Z. and Y.W. wrote the manuscript. All authors contributed to data interpretation and the discussion of results in the manuscript.

## Funding

This work was supported by the National Natural Science Foundation of China (82172820), the Natural Science Foundation of Shanghai (22ZR1466200), the Clinical Research Special Funding of the Shanghai Municipal Health Commission (202340112), the Fundamental Research Funds for the Central Universities (22120260208 and 22120250457), the Key Disciplines Group Construction Project of Shanghai Pudong New Area Health Commission (PWZxq2022‐10), the New Quality Clinical Specialty Program of High‐end Medical Disciplinary Construction in Shanghai Pudong New Area (2026‐PWXZ‐07) and the Key Discipline Construction Project of Shanghai East Hospital (2024‐DFZD‐003S).

## Conflicts of Interest

The authors declare no conflicts of interest.

## Supporting information




**Supporting File**: advs77209‐sup‐0001‐SuppMat.docx.

## Data Availability

The data that supports the findings of this study are available in the supplementary material of this article.
