# Supplementary material for: Lactate‐Induced K370 Lactylation of STING Inhibits STING‐TBK1 Signaling and Dampens Anti‐Tumor Immunity
Source: Adv Sci (Weinh). 2026 Aug 15:e77209. Online ahead of print. doi: 10.1002/advs.77209 (PMC13477259; doi:10.1002/advs.77209)
Supplement: Supplementary file 1 [file ADVS-9999-e77209-s001.docx]

**Fig. S1.** **Lactate-mediated limitation of STING pathway activation in GBM.**

**(A)** Kaplan-Meier analysis of the diseases free survival (DFS) comparing high and low LDHA expression in GBM patients (n = 45). Patients were stratified into two groups according to the median LDHA expression level. *P*-values by the log-rank test.

**(B)** Kaplan-Meier analysis of the overall survival (OS) comparing high and low LDHA expression from the TCGA-GBM dataset (n = 142). The TCGA-GBM dataset refers to the Glioblastoma Multiforme cohort from The Cancer Genome Atlas.

**(C)** Representative immunofluorescence images of STING and p-STING (S366) in GBM tumor tissues and adjacent normal tissues. Scale bar: 50 μm.

**(D)** Quantification of the p-STING/STING immunofluorescence intensity ratio in GBM tumor tissues and adjacent normal tissues. Data are shown as mean ± SD (n = 3). *P*-values by two-tailed t-test. ***P < 0.001.

**(E)** Protein expression of Pan-Lac and p-STING in GBM tumor tissues with high or low LDHA expression (n = 18 per group). *P*-values by two-tailed t-test. ***P < 0.001.

**(F)** Quantification of the STING/β-Actin and p-STING/STING protein expression ratios in U251 GBM cell lines under different treatment conditions. Data are shown as mean ± SD (n = 3). *P*-values by one-way ANOVA. *P < 0.05, **P < 0.01.

**(G)** Representative immunofluorescence images of Ki67 and IFN-β in tumor tissues in GBM patients. Scale bar: 50 μm.

**(H)** Representative immunofluorescence images of CD68, CD206, and FOXP3 in tumor tissues in GBM patients. Scale bar: 50 μm.

**(I)** Quantification of Ki67 or IFN-β immunofluorescence staining in tumor tissues in GBM patients. Data are shown as mean ± SD (n = 3), and two-tailed t-test determines the *P*-values. ***P < 0.001.

**(J)** Quantification of CD68 and CD206 or FOXP3 immunofluorescence staining in tumor tissues in GBM patients. Data are shown as mean ± SD, (n = 3), and two-tailed t-test determines the *P*-values. **P < 0.01, ***P < 0.001.

**Fig. S2.** **Additional evidence supporting the role of STING K370 lactylation in regulating TBK1 binding.**

**(A)** Representative mass spectrometry spectrum identifying K338 as a lactylation site on STING.

**(B)** Co-immunoprecipitation and western blot analysis of STING K370 lactylation in HEK293T cells expressing Flag-tagged STING-WT or STING-K338R, with or without MG132 treatment (15 μM for 6 h before harvesting), using a site-specific anti-STING-K370la antibody.

**(C)** Western blot analysis of STING S366 phosphorylation in HEK293T cells expressing Flag-tagged STING-WT, STING-K338R, or STING-K370R following stimulation with cGAMP (100 nM) for 6 h, in the presence or absence of MG132 treatment (15 μM for 6 h before harvesting).

**(D)** Co-immunoprecipitation and western blot analysis of total STING lactylation and STING K370 lactylation in HEK293T cells co-expressing Flag-tagged STING and Myc-tagged SIRT1–SIRT7 as indicated.

**(E)** Coomassie Brilliant Blue staining of purified GST-STING-WT and GST-STING-K370R proteins used for in vitro lactylation assays.

**(F)** Co-immunoprecipitation and western blot analysis of the interaction between STING and TBK1 in HEK293T cells treated with NALA (25 mM) or LDHAi (15 mM) for 24 h.

**(G)** Co-immunoprecipitation and western blot analysis of the interaction between TBK1 and STING in HEK293T cells treated with NALA (25 mM) or LDHAi (15 mM) for 24 h, followed by stimulation with cGAMP (100 nM) for 6 h before harvesting.

**(H)** Co-immunoprecipitation and western blot analysis of STING K370 lactylation and STING S366 phosphorylation in HEK293T cells expressing Flag-tagged STING-WT following shRNA-mediated knockdown of CBP, with or without treatment with the TBK1 inhibitor GSK8612 (5 μM) for 24 h, followed by stimulation with cGAMP (100 nM) for 6 h before harvesting.

**Fig. S3.** **Lactylation regulates STING ubiquitination, protein stability, and intermolecular interactions.**

**(A-B)** Co-immunoprecipitation and western blot analysis of STING ubiquitination in HEK293T cells co-transfected with HA-tagged ubiquitin (Ub) and Flag-tagged STING (HA-MCS served as the empty vector control). After 24 h of transfection, cells were treated with NALA (25 mM) (A) or LDHAi (15 mM) (B) for 24 h, followed by MG132 treatment (15 μM) for 6 h before harvesting.

**(C)** Native-PAGE and SDS-PAGE immunoblot analysis of STING oligomerization and expression in U251 cells expressing Flag-tagged STING-WT or STING-K370R following SR-717 stimulation (10 μM) for 24 h.

**(D)** Western blot analysis of STING protein stability in U251 cells treated with increasing concentrations of NALA (15 and 25 mM) for 24 h, followed by CHX (100 ng/mL) treatment for 6 h before harvesting.

**(E)** Western blot analysis of STING protein stability in U251 cells treated with NALA (25 mM) for 24 h, followed by CHX (100 ng/mL) treatment for the indicated durations (6 and 12 h) before harvesting.

**(F)** Western blot analysis of STING protein stability in U251 cells treated with NALA (25 mM) or LDHAi (15 mM) for 24 h, followed by CHX (100 ng/mL) treatment for 6 h before harvesting.

**(G)** Western blot analysis of STING protein stability in patient-derived GBM cells (PDCs) with or without shRNA-mediated knockdown of LDHA, followed by CHX (100 ng/mL) treatment for the indicated durations (0, 6, and 12 h).

**(H)** Western blot analysis of STING protein stability in patient-derived GBM cells (PDCs) with or without shRNA-mediated knockdown of CBP, followed by CHX (100 ng/mL) treatment for the indicated durations (0, 6, and 12 h).

**(I)** Co-immunoprecipitation and western blot analysis of STING lactylation and STING-TBK1 interaction in U251 cells expressing Flag-tagged STING-WT or STING-K370R, with or without MG132 treatment (15 μM for 6 h before harvesting).

**(J)** Co-immunoprecipitation and western blot analysis of STING lactylation and STING interactions with TBK1 and SEC24A in U251 cells expressing Flag-tagged STING-WT or STING-K370R, with or without MG132 treatment (15 μM for 6 h before harvesting), followed by stimulation with SR-717 (10 μM) for 24 h.

**(K)** Co-immunoprecipitation and western blot analysis of the interaction between TBK1 and TMEM192-STING-WT or TMEM192-STING-K370R in U251 cells treated with MG132 (15 μM), or MG132 (15 μM) plus chloroquine (CQ, 10 μM), for 6 h before harvesting.

**Fig. S4.** **Celastrol suppresses lactate production and global protein lactylation in GBM cells.**

**(A)** Kaplan-Meier analysis of the overall survival (OS) comparing high and low STING-K370la expression in GBM patients (n = 39). Patients were stratified into two groups according to the median STING-K370la expression level. *P*-values by the log-rank test.

**(B)** Kaplan-Meier analysis of the disease-free survival (DFS) comparing high and low STING-K370la expression in GBM patients (n = 39). Patients were stratified into two groups according to the median STING-K370la expression level. *P*-values by the log-rank test.

**(C)** Mutational concordance and recurrent genomic alterations identified in primary GBM tumors and the corresponding matched PDCs and PDOs. Variant allele frequencies (VAFs) are color-coded as indicated.

**(D)** Representative H&E staining and immunofluorescence images of S100β (green) and GFAP (red) in primary GBM tumor tissues and matched PDOs. Scale bars: 25 μm.

**(E)** Representative immunofluorescence images of CD31 (green) in primary GBM tumor tissues and matched PDOs. Scale bars: 25 μm.

**(F-H)** Cell viability of the indicated GBM cell lines following treatment with Celastrol (Cel) for 48 h, as determined by CCK-8 assay.

**(I-K)** Western blot analysis of LDHA expression and global protein lactylation levels in the indicated GBM cell lines treated with Cel (1 or 2 μM) for 48 h.

**Fig. S5 LDHA inhibition enhances SR-717–induced STING pathway activation in patient-derived GBM cells.**

**(A-B)** Quantification of the p-STING/STING, p-TBK1/TBK1, and p-IRF3/IRF3 protein expression ratios in patient-derived GBM cells (PDCs) under the indicated treatment conditions. Data are shown as mean ± SD (n = 3). *P*-values by one-way ANOVA. *P < 0.05, ***P < 0.001.

**(C)** Co-immunoprecipitation and western blot analysis of STING lactylation in PDCs treated with SR-717 (5 μM) in the presence or absence of Cel (2 μM) for 48 h.

**(D)** Cell viability of SVGp12 cells treated with Cel (2 μM) and SR-717 (5 μM) for 48 h, as determined by CCK-8 assay. Data are shown as mean ± SD (n = 4). *P*-values by one-way ANOVA.

**(E)** Western blot analysis of STING S366 phosphorylation in PDCs following shRNA-mediated knockdown of LDHA and stimulation with SR-717 (5 μM) for 48 h.

**(F-G)** Western blot analysis of STING S366 phosphorylation in PDCs treated with the LDHA inhibitors FX-11 (25 μM) or Stiripentol (100 μM), together with SR-717 (5 μM) for 48 h.

**(H)** Representative immunofluorescence images of IFN-β in PDCs under the indicated treatment conditions. Scale bars: 10 μm.

**(I)** Quantification of IFN-β fluorescence intensity in PDCs. Data are shown as mean ± SD (n = 3). *P*-values by one-way ANOVA. ***P < 0.001.

**Fig. S6 Combined LDHA inhibition and STING activation suppress PDO growth and enhance antitumor responses.**

**(A-B)** Representative H&E staining images and morphological assessment of patient-derived GBM organoids (PDOs) treated with Celastrol (Cel, 2 μM) and SR-717 (5 μM) for 72 h. Scale bars: 25μm, 100 μm, top to bottom.

**(C-D)** Three-dimensional viability of PDOs following treatment with Cel (2 μM) and SR-717 (5 μM) for 72 h, as determined by the CellTiter-Glo (CTG) luminescence assay. Data are shown as mean ± SD (n = 4). *P*-values by one-way ANOVA. ***P < 0.001.

**(E)** Representative immunofluorescence images of Ki67 in PDOs following the indicated treatment regimenss. Scale bars: 25 μm.

**(F)** Quantification of the percentage of Ki67-positive cells in PDOs following the indicated treatment regimens. Data are shown as mean ± SD (n = 3). *P*-values by one-way ANOVA. **P < 0.01, ***P < 0.001.

**(G)** Representative immunofluorescence images of IFN-β in PDOs following the indicated treatment regimens. Scale bars: 25 μm.

**(H)** Quantification of the percentage of IFN-β-positive cells in PDOs following the indicated treatment regimens. Data are shown as mean ± SD (n = 3). *P*-values by one-way ANOVA. *P < 0.05, ***P < 0.001.

**Fig. S7.** **Combined LDHA inhibition, STING activation, and PD-1 blockade enhance antitumor responses and reshape the immune microenvironment in PDOs.**

**(A)** Representative immunofluorescence images of CD68 (green) and CD206 (red) in primary GBM tumor tissues and matched PDOs. Scale bars: 25 μm.

**(B)** Representative immunofluorescence images of FOXP3 (green) in primary GBM tumor tissues and matched PDOs. Scale bars: 25 μm.

**(C-E)** Representative H&E staining images and morphological assessment of PDOs treated with Celastrol (Cel, 2 μM), SR-717 (5 μM), and anti-PD-1 antibody for 5 days. Scale bars: 25μm, 100 μm, top to bottom.

**(F-H)** Representative immunofluorescence images of Ki67 (green) and IFN-β (red) in PDOs following the indicated treatment regimens. Scale bars: 25 μm.

**(I-K)** Three-dimensional viability of PDOs treated with Cel (2 μM), SR-717 (5 μM), and anti-PD-1 antibody for 5 days, as determined by the CellTiter-Glo (CTG) luminescence assay. Data are shown as mean ± SD (n = 4). *P*-values by one-way ANOVA. ***P < 0.001.

**(L-M)** Representative immunofluorescence images of CD68, CD206, and CD86 in PDOs subjected to the indicated treatment regimens, including Cel (2 μM), SR-717 (5 μM), anti-PD-1 antibody, and anti-IFNAR antibody, for 5 days. Scale bars: 25 μm.

**(N-O)** Quantification of the percentages of CD68⁺CD206⁺ and CD68⁺CD86⁺ cells in PDOs following the indicated treatment regimens. Data are shown as mean ± SD (n = 3). *P*-values by one-way ANOVA. *P < 0.05, **P < 0.01, ***P < 0.001.

**Fig. S8. Cel potentiates immune microenvironment remodeling induced by combined STING activation and PD-1 blockade in PDOs.**

**(A-B)** Representative immunofluorescence images of CD68 and CD206 or CD86 in PDOs following the indicated treatment regimens, including SR-717 (5 μM) plus anti-PD-1 antibody, or Cel (2 μM) combined with SR-717 (5 μM) and anti-PD-1 antibody, for 5 days. Scale bars: 25 μm.

**(C-D)** Quantification of the percentages of M2-like (CD68⁺CD206⁺) and M1-like (CD68⁺CD86⁺) tumor-associated macrophages (TAMs) in PDOs following the indicated treatment regimens. Data are shown as mean ± SD (n = 3). *P*-values by one-way ANOVA. **P < 0.01, ***P < 0.001.

**(E-F)** Representative immunofluorescence images of FOXP3 and CD8 in PDOs following the indicated treatment regimens. Scale bars: 25 μm.

**(G-H)** Quantification of the percentages of FOXP3⁺ and CD8⁺ T cells in PDOs following the indicated treatment regimens. Data are shown as mean ± SD (n = 3). *P*-values by one-way ANOVA. **P < 0.01, ***P < 0.001.

**Fig. S9. Flow cytometry gating strategy and GL261-hSTING model characterization.**

**(A)** Representative flow cytometry gating strategy for the identification of CD45⁺CD3⁺CD8⁺ T cells in patient-derived GBM organoids (PDOs).

**(B)** Western blot analysis of mouse STING (mSTING) and human STING (hSTING) expression in GL261-hSTING cells.

**(C)** Activation of the STING signaling pathway was assessed by western blotting in GL261-hSTING cells treated with Celastrol (Cel, 2 μM) and SR-717 (5 μM) for 48 h.

**Supplementary Table S1**

**Representative Patient Sample Information**

| Patient ID | Gender | Age | Diagnosis | Primary/Recurrent | IDH |
| --- | --- | --- | --- | --- | --- |
| 7263 | female | 59 | WHO IV,  Glioblastoma | Primary | Wild type |
| 1641 | male | 59 | WHO IV,  Glioblastoma | Primary | Wild type |
| 5157 | male | 54 | WHO IV,  Glioblastoma | Primary | Wild type |
| 7283 | male | 74 | WHO IV,  Glioblastoma | Primary | Wild type |
| 7241 | male | 69 | WHO IV,  Glioblastoma | Primary | Wild type |
| 7220 | male | 75 | WHO IV,  Glioblastoma | Primary | Wild type |
| 6864 | male | 77 | WHO IV,  Glioblastoma | Primary | Wild type |
| 6981 | female | 59 | WHO IV,  Glioblastoma | Primary | Wild type |
| 7667 | female | 61 | WHO IV,  Glioblastoma | Primary | Wild type |

**Supplementary Table S2**

**Key Resources Table**

| Reagent or Resource | Source | Identifier |
| --- | --- | --- |
| Antibodies | | |
| Anti-LDHA | Cell Signaling Technology | Cat# 3582S; RRID: AB_2066887 |
| Anti-human STING | R&D Systems | Cat# AF6516; RRID: AB_10718401 |
| Anti-STING | Cell Signaling Technology | Cat# 13647S; RRID: AB_2732796 |
| Anti-mouse STING | Abcam | Cat# ab288157; RRID: AB_3086730 |
| Anti-Phospho-STING (Ser366) | Cell Signaling Technology | Cat# 50907S; RRID: AB_2827656 |
| Anti-TBK1 | Cell Signaling Technology | Cat# 38066S; RRID: AB_2827657 |
| Anti-Phospho-TBK1(Ser172) | Cell Signaling Technology | Cat# 5483S; RRID: AB_10693472 |
| Anti-IRF3 | Abcam | Cat# ab68481; RRID: AB_11155653 |
| Anti-Phospho-IRF3 (Ser396) | Cell Signaling Technology | Cat# 29047S; RRID: AB_2773013 |
| Anti-Interferon beta | Abcam | Cat# ab85803; RRID: AB_10674887 |
| Anti-Ki67 | Abcam | Cat# ab16667; RRID: AB_302459 |
| Anti-CD68 | Abcam | Cat# ab955; RRID: AB_307338 |
| Anti-CD86 | Cell Signaling Technology | Cat# 91882S; RRID: AB_2797422 |
| Anti-CD206 | Cell Signaling Technology | Cat# 24595; RRID: AB_2892682 |
| Anti-CD8 alpha | Abcam | Cat# ab237709; RRID: AB_2892677 |
| Anti-FOXP3 | Thermo Fisher Scientific | Cat# 14-4777-82; RRID: AB_467556 |
| Anti- L-Lactyl Lysine | PTM BIO | Cat# PTM-1401RM; RRID: AB_2942013 |
| Anti-CBP | Cell Signaling Technology | Cat# 7389S RRID: AB_2616020 |
| Anti-SEC24A | Proteintech | Cat# 15958-1-AP; RRID: AB_2186118 |
| Anti-TMEM192 | Abcam | Cat# ab186737; RRID: AB_3095637 |
| Anti-Calreticulin | Abcam | Cat# ab2907; RRID: AB_303402 |
| Anti-GM130 | BD Biosciences | Cat# 610822; RRID: AB_398142 |
| Anti-CD31 | Cell Signaling Technology | Cat# 3528S; RRID: AB_2160882 |
| Anti-S100 beta | Sigma Aldrich | Cat# S2532; RRID: AB_477499 |
| Anti-GFAP | Cell Signaling Technology | Cat# 80788S; RRID: AB_2799963 |
| Anti-STING-K370la | TENZOBIO | Cat# N/A |
| Anti-HA Tag | Cell Signaling Technology | Cat# 3724S; RRID: AB_1549585 |
| Anti-Flag Tag | Cell Signaling Technology | Cat# 14793S; RRID: AB_2572291 |
| Anti-Flag Tag | Sigma Aldrich | Cat# F1804; RRID: AB_262044 |
| Anti-beta Actin | Affinity | Cat# AF7018; RRID: AB_2839420 |
| Chemicals, peptides, and recombinant proteins | | |
| Sodium L-lactate | Sigma Aldrich | Cat# 71718 |
| Sodium oxamate | MedChemExpress | Cat# HY-W013032A |
| Celastrol | TargetMol | Cat# T3028 |
| SR-717 | TargetMol | Cat# T8655 |
| 2',3'-cGAMP | MedChemExpress | Cat# HY-100564 |
| MG132 | TargetMol | Cat# T2154 |
| Cycloheximide | TargetMol | Cat# T1225 |
| Chloroquine | TargetMol | Cat# T8689 |
| Lactyl-CoA | TargetMol | Cat# TXB-00809 |
| GSK8612 | TargetMol | Cat# T5540 |
| FX-11 | TargetMol | Cat# T15362 |
| Stiripentol | TargetMol | Cat# T4498 |
| In vivo anti-mouse PD-1 | TargetMol | Cat# T78269 |
| Anti-HA-agarose beads | Abclonal | Cat# AE059 |
| Anti-Flag-agarose beads | GenScript | Cat# L00432 |
| GST-tag Purification Resin | Beyotime | Cat# P2250 |
| HA-peptide | Beyotime | Cat# P9808 |
| In vivo anti-human PD-1 | BioXcell | Cat# BE0188 |
| In vivo anti-human IFNAR | BioXcell | Cat# SIM0022 |
| Human TruStain FcX™ | BioLegend | Cat# 422301 |
| FITC anti-human CD45 | BioLegend | Cat# 304006 |
| APC anti-human CD3 | BioLegend | Cat# 300412 |
| PE anti-human CD8a | BioLegend | Cat# 300908 |
